# Analysis of Gene Order Conservation in Eukaryotes Identifies Transcriptionally and Functionally Linked Genes

**DOI:** 10.1371/journal.pone.0010654

**Published:** 2010-05-14

**Authors:** Marcela Dávila López, Juan José Martínez Guerra, Tore Samuelsson

**Affiliations:** 1 Department of Medical Biochemistry and Cell Biology, Institute of Biomedicine, Sahlgrenska Academy at University of Gothenburg, Göteborg, Sweden; 2 Departmento de Química, Centro de Ciencias Básicas, Universidad Autónoma de Aguascalientes, Aguascalientes, Aguascalientes, Mexico; American Museum of Natural History, United States of America

## Abstract

The order of genes in eukaryotes is not entirely random. Studies of gene order conservation are important to understand genome evolution and to reveal mechanisms why certain neighboring genes are more difficult to separate during evolution. Here, genome-wide gene order information was compiled for 64 species, representing a wide variety of eukaryotic phyla. This information is presented in a browser where gene order may be displayed and compared between species. Factors related to non-random gene order in eukaryotes were examined by considering pairs of neighboring genes. The evolutionary conservation of gene pairs was studied with respect to relative transcriptional direction, intergenic distance and functional relationship as inferred by gene ontology. The results show that among gene pairs that are conserved the divergently and co-directionally transcribed genes are much more common than those that are convergently transcribed. Furthermore, highly conserved pairs, in particular those of fungi, are characterized by a short intergenic distance. Finally, gene pairs of metazoa and fungi that are evolutionary conserved and that are divergently transcribed are much more likely to be related by function as compared to poorly conserved gene pairs. One example is the ribosomal protein gene pair L13/S16, which is unusual as it occurs both in fungi and alveolates. A specific functional relationship between these two proteins is also suggested by the fact that they are part of the same operon in both eubacteria and archaea. In conclusion, factors associated with non-random gene order in eukaryotes include relative gene orientation, intergenic distance and functional relationships. It seems likely that certain pairs of genes are conserved because the genes involved have a transcriptional and/or functional relationship. The results also indicate that studies of gene order conservation aid in identifying genes that are related in terms of transcriptional control.

## Introduction

Recombination events result in shuffling of genes in genomes during evolution. In bacteria many genes are organized in operons, and as a result shuffling of genes is constrained. Eukaryotes, however, are not subject to this restriction and gene order is to a large extent random. Thus, if we compare two eukaryotic genomes that are only distantly related, it is very unlikely that two genes are in the same order in the two species.

Nevertheless gene order is not completely random in eukaryotes. A number of associated factors have been identified [1]. Thus, genes of similar expression tend to cluster more commonly than expected by chance [2], [3], [4], [5], [6]. There is also evidence that functionally related genes tend to cluster. Thus, a significant number of genes encoding subunits of stable complexes are located within close proximity of each other [7], [8], [9]. Moreover, genes from the same metabolic pathway tend to cluster [10]. An intriguing case is a gene cluster in *Saccharomyces cerevisiae* that enables the use of allantoin as nitrogen source. In this case an evolution is observed where genes that were previously scattered around the genome became relocated to a single site in an ancestor of *S. cerevisiae* and *Saccharomyces castellii* [11]. Another example of evolution involving relocation of genes is a cluster of *Drosophila* genes [12]. There are also cases of gene duplication where the resulting paralogous genes are clustered. Classic examples include the vertebrate beta-globin locus, as well as the Hox and histone genes.

Another factor associated with non-random gene order is intergenic distance. It has been shown that in yeasts this is a strong predictor of gene order conservation [13]. In mammalian genomes gene pairs are abundant that have a short intergenic distance and where the genes are divergently transcribed [8], [9], [14], [15], [16], [17].

Divergently transcribed genes with an intergenic region less than 1000 base pairs are assumed to have a promoter region (“bidirectional promoter”) with sequence elements shared between the two genes [14]. The genes in such pairs often encode two different peptide subunits that share structural and functional characteristics (for instance collagen [18]), or that are involved in the same cellular pathway (such as TAP1/LMP2 [19]). Bidirectional promoters are often associated with genes that function in DNA repair. Therefore, there is potentially a relationship between such gene pairs and cancer. Indeed, it was recently shown that among human genes implicated in breast and ovarian cancer bidirectional promoters are enriched [9].

In cases where we observe a strong gene order conservation in eukaryotes it seems likely that genes are related in terms of transcriptional control. In the case of divergently transcribed genes they could share promoter and transcriptional regulatory signals and as a result be co-expressed [8], [18]. Conversely, they could be antiregulated, i.e. when one gene is turned on the other is turned off, and vice versa [20], [21]. In such cases we expect important regulatory elements to be found in the intergenic region. Hence, regulation may be exerted through short-range effects. Regulation of expression may also be exerted at a higher level through chromatin remodeling. Neighboring genes may be prevented from expression by histone modification and gene expression may depend on DNA methylation. Methylation of CpG island promoter regions is a common feature of human neoplasia. Also divergently transcribed genes with bidirectional promoters are controlled by promoter methylation as in the case of tumor suppressor genes [22], [23], [24]. Therefore, studies of gene order conservation may reveal important clues as to transcriptional control mechanisms.

So far, non-random gene order has been studied in a limited number of eukaryotic species. In order to identify parameters important for non-random gene order we have here taken a more systematic comparative genomics approach by considering 64 different eukaryotic species from a wide variety of eukaryotic phyla. We focused on a set of parameters of interest to gene order; relative transcription direction, intergenic distance, and functional relationships as inferred from gene ontology and we examined the relationship of these three parameters to evolutionary conservation.

## Results

### Gene order information

**Figure 1:**
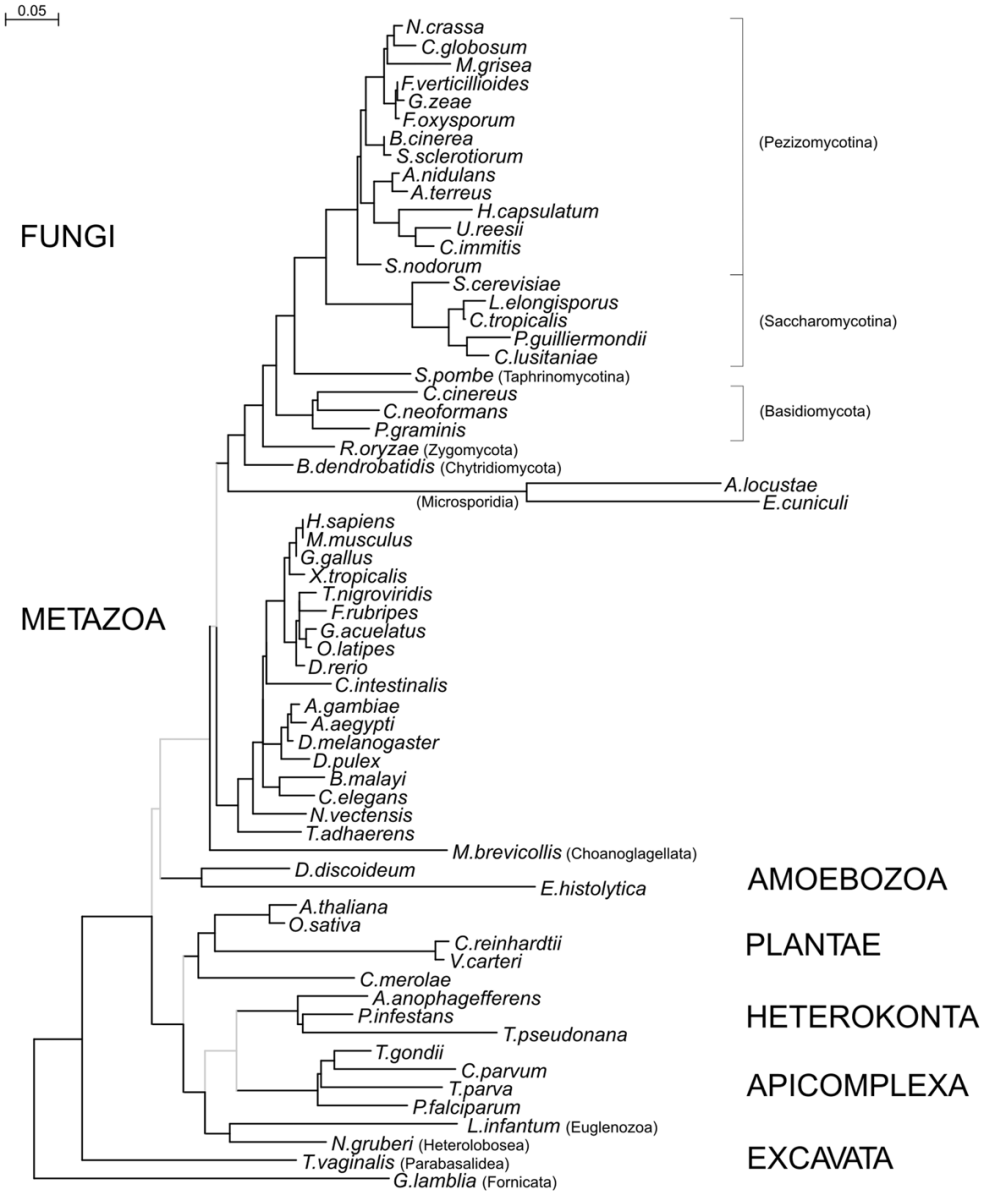
Phylogenetic tree of species used in this study. Tree was constructed by parsimony analysis of concatenated α-tubulin, β-tubulin, actin and the elongation factor 1-alpha (EF-1α) amino acid sequences, as further described under “Materials and Methods”.

In order to examine eukaryotic gene order we collected information on protein encoding genes from 64 different eukaryotic genomes, representing all major phylogenic groups where genome sequence is available. A phylogenetic tree of these is shown in Figure 1. The branch lengths of this tree were used in order to estimate the evolutionary distance between two species as described further below.

In order to be able to compare gene order in different species orthologues were identified with OrthoMCL [25]. The OrthoMCL clustering generated a total of 71,219 clusters, involving 652,857 proteins (about 80% of all proteins). Proteins were also characterized with the help of Pfam domains and architectures [26] as well as with respect to gene ontology (GO) (for details see under “Materials and Methods”). Using the Pfam architecture classification, we were able to group 458,597 proteins, i.e. a smaller number as compared to the OrthoMCL clustering method. Furthermore, Pfam classification typically resulted in groups with a larger number of proteins as compared to the OrthoMCL clustering. For instance, in the case of histones, 12 clusters were obtained with OrthoMCL, each cluster with a different type of histone (such as H1, H2A, H2B, H3 and H4), while in Pfam all these families are collected in only one group.

All pairs of neighboring genes were classified according to their relative orientation. Thus, they could be transcribed on the same strand (→ →), or on opposite strands in a ‘head to head’ (← →) or ‘tail to tail’ (→ ←) fashion (Table S1). These three categories of gene pairs will be referred to as co-directionally, divergently and convergently transcribed, respectively.

In order to compare the evolutionary conservation of gene pairs, we used a measure of conservation which is the sum of branch lengths of the subtree involving the respective species. In general, a larger evolutionary distance between gene pairs were observed in the case of Pfam grouping, because of the differences in grouping proteins with OrthoMCL and Pfam as mentioned above, where OrthoMCL gave rise to a larger number of clusters. Information about the most conserved gene pairs is available in Table S2.

**Figure 2:**
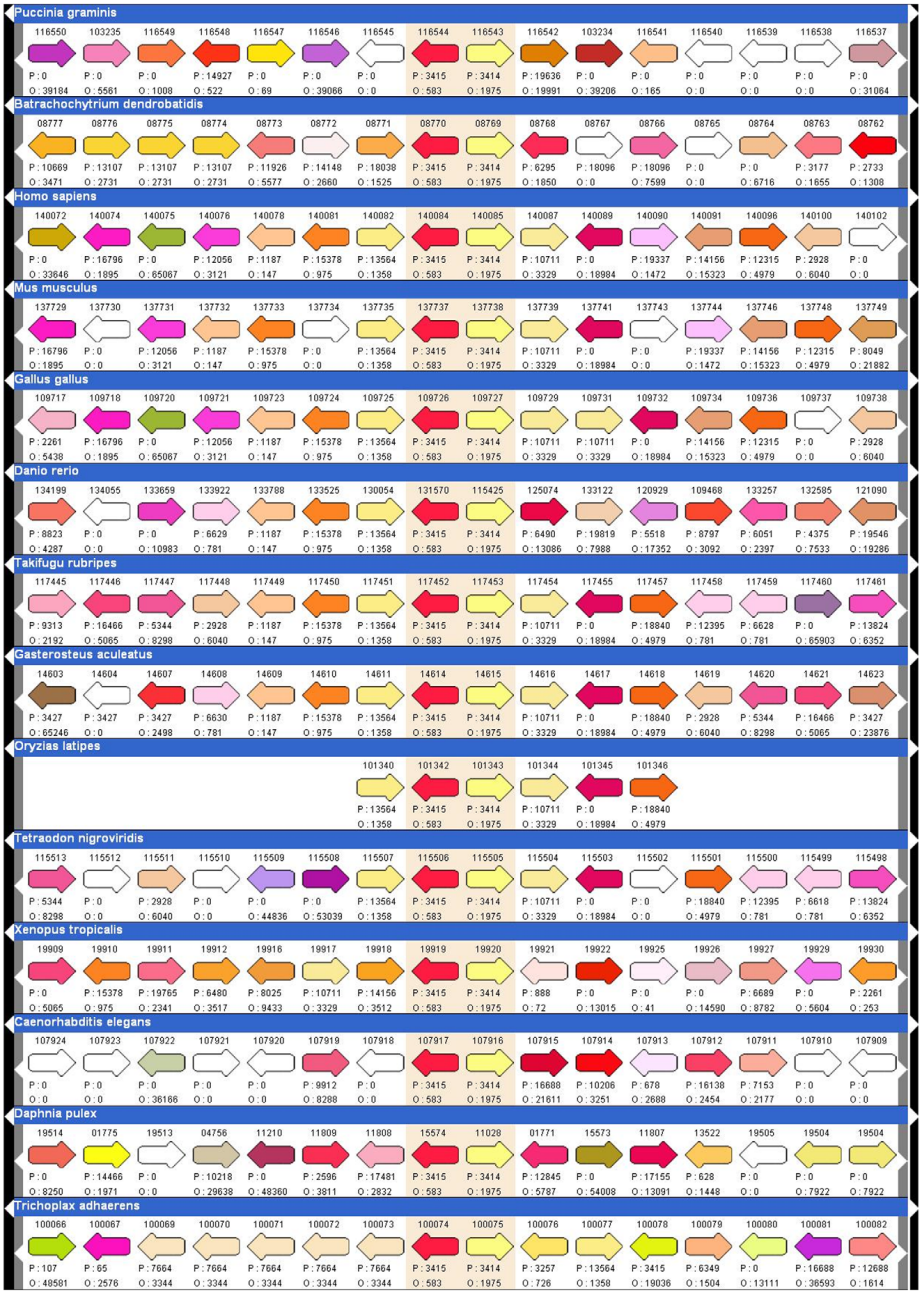
Eukaryotic Gene Order Browser (eGOB). Genomic context of organisms that share a divergently transcribed pair of the heat shock proteins Hsp10 (red) and Hsp60 (yellow) as seen through the Eukaryotic Gene Order Browser (http://egob.biomedicine.gu.se). Arrows indicate the relative directions of genes. Homologous sequences, i.e. protein sequences that belong to the same cluster as defined in this case by OrthoMCL, are in the same colour.

Information regarding relative gene order may be accessed at the “eukaryotic Gene Order Browser” (eGOB) at http://egob.biomedicine.gu.se. This browser allows a user to view any eukaryotic gene and its environment in different species. A gene or protein of interest may be identified by performing queries based on Uniprot-Swissprot IDs or description of protein function. In addition, Pfam domains may be queried as well as GO terms and it is also possible to identify a protein based on a BLAST search. Queries may be restricted to specific species or phylogenetic groups and evolutionary conserved pairs of adjacent genes may also be identified. An example screen-shot in Figure 2 shows two adjacent, divergently transcribed genes, the 60 kDa heat shock protein (HSPD1) and the 10 kDa heat shock protein (HSPE1) in different species. Genes are represented by arrows, which denote the relative direction of transcription. Each gene is color-coded according to the OrthoMCL/Pfam cluster to which it belongs. The example in Figure 2 illustrates the ability to compare gene order in different species.

With available information on pairs of adjacent genes we now examined factors that are related to a non-random gene order. In particular, we focused on evolutionary conservation of gene pairs as related to relative transcriptional direction, intergenic distance, and functional relationships as deduced from gene ontology.

### Conserved gene pairs are divergently or co-directionally transcribed

For a majority of species examined here, co-directional pairs are found at a frequency of about 50% (Table S1, Figure S1). This is what to be expected if the direction of genes is random. However, there are a few species that are unusual as the distribution of gene direction seems less random. This applies to *Monosiga brevicollis*, *Cyanidioschyzon merolae*, *Thalassiosira pseudonana* and *Cryptococcus neoformans* (Figure S1.) A special case is *Leishmania*, where 98% of the gene pairs are arranged in a co-directional fashion, but this is to be expected from the polycistronic gene organization found in Leishmania and other related kinetoplastids [27], [28].

**Figure 3:**
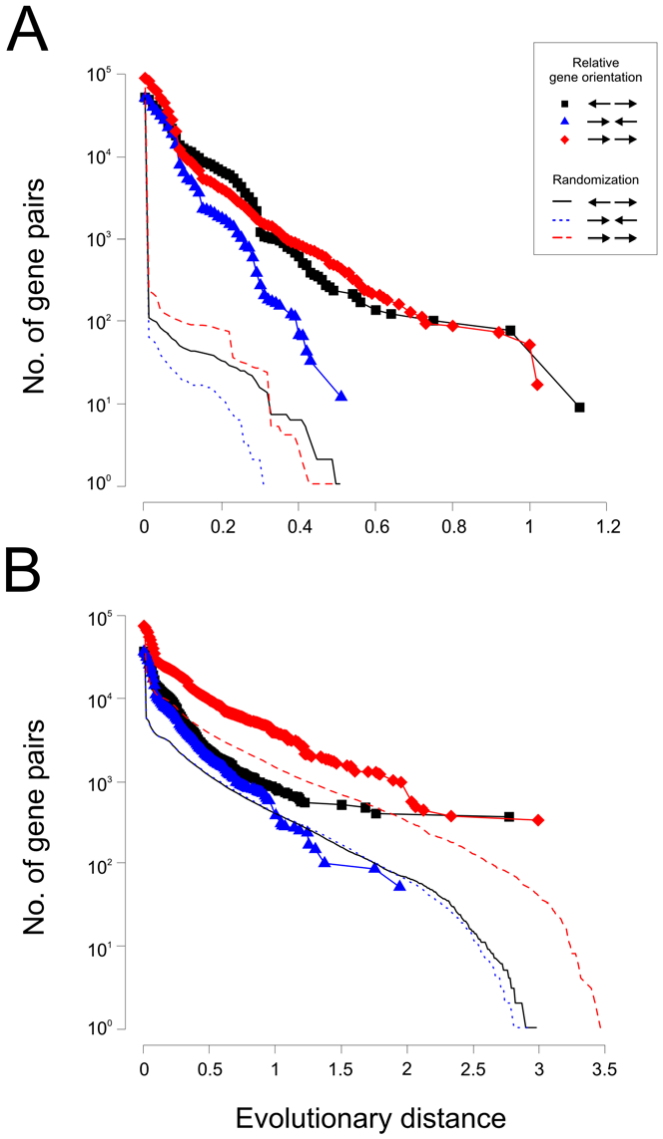
Evolutionary conservation and relative gene orientation. For a range of evolutionary distances within an interval of 0.01 units the number of gene pairs corresponding to a certain relative gene orientation was calculated and plotted. Gene orientation considered were divergent (← →), convergent (→ ←) and co-directional (→→). Cumulative counts of gene pairs are shown. Randomized counts were obtained by shuffling for every species the identities of OrthoMCL clusters or Pfam groups. Based on these results of randomizations it would seem that the probability of finding a pair of genes with the same relative orientation in at least two different species by chance only is approximately 0.002–0.01. A. Genes clustered using OrthoMCL. B. Genes grouped on the basis of Pfam architectures.

For prokaryotic genomes it has been shown that pairs of divergently transcribed genes, as well as co-directional pairs, are conserved across evolutionary distant species in a manner which is not expected by chance [29]. To examine whether such a relationship applies to eukaryotic species we analyzed the number of gene pairs as a function of evolutionary distance, using both OrthoMCL (Figure 3A) and Pfam grouping (Figure 3B). A result similar to that of prokaryotes was indeed obtained. When considering the highly conserved gene pairs, those that are divergently and co-directionally transcribed occur at high frequency.

### Evolutionary conservation of gene pairs with a short intergenic distance

Average intergenic distances for all species examined were calculated (Table S3). It is well known that metazoa in general have much larger intergenic distances than fungi but we now provide detailed information on this. Most protozoa have very short intergenic distances, in particular Dictyostelium, Entamoeba, Giardia and Cryptosporidium.

**Figure 4:**
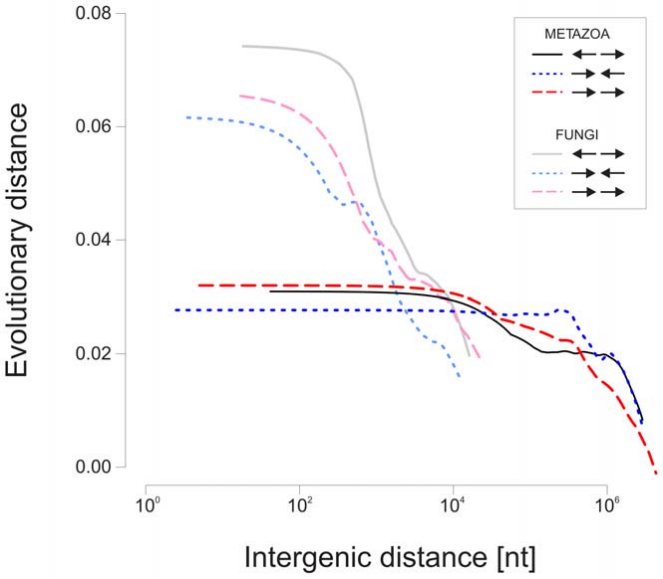
Relationship between intergenic distance and evolutionary conservation within the phylogenetic groups Metazoa and Fungi. For all gene pairs present in more than one species a measure of evolutionary conservation was calculated based on the species involved as described under Materials and Methods. Lowess regression lines are shown. For calculation of evolutionary conservation groups only species within the respective groups (i.e Metazoa and Fungi) were considered. For reference, the mean values of intergenic distances for the divergently (← →), convergently (→ ←) and co-directionally (→→) transcribed gene pairs are 34912, 34165 and 22923 for Metazoa and 1343, 688 and 1230 for Fungi.

Intergenic distance has previously been identified as a strong predictor of gene order conservation in fungi [13]. To examine the relationship of conservation and intergenic distance for the eukaryotes analyzed here we determined for each pair of adjacent genes occurring in at least two different species the mean value for the intergenic distances involved and at the same time a measure of evolutionary conservation was calculated for the species considered. Each of the three different gene orientation categories was analyzed separately. The results for Metazoa and Fungi in Figure 4 show that as the intergenic distance decreases the measure of evolutionary conservation increases, reaching an optimum at 100–1000 nt for fungi and in the order of 10,000 nt for metazoa. A comparison of the three relative gene orientations shows that divergently transcribed genes in fungi stand out as being particularly strongly conserved at a shorter intergenic distance. This effect is not observable for other phylogenetic groups such as metazoa (Figure 4).

**Figure 5:**
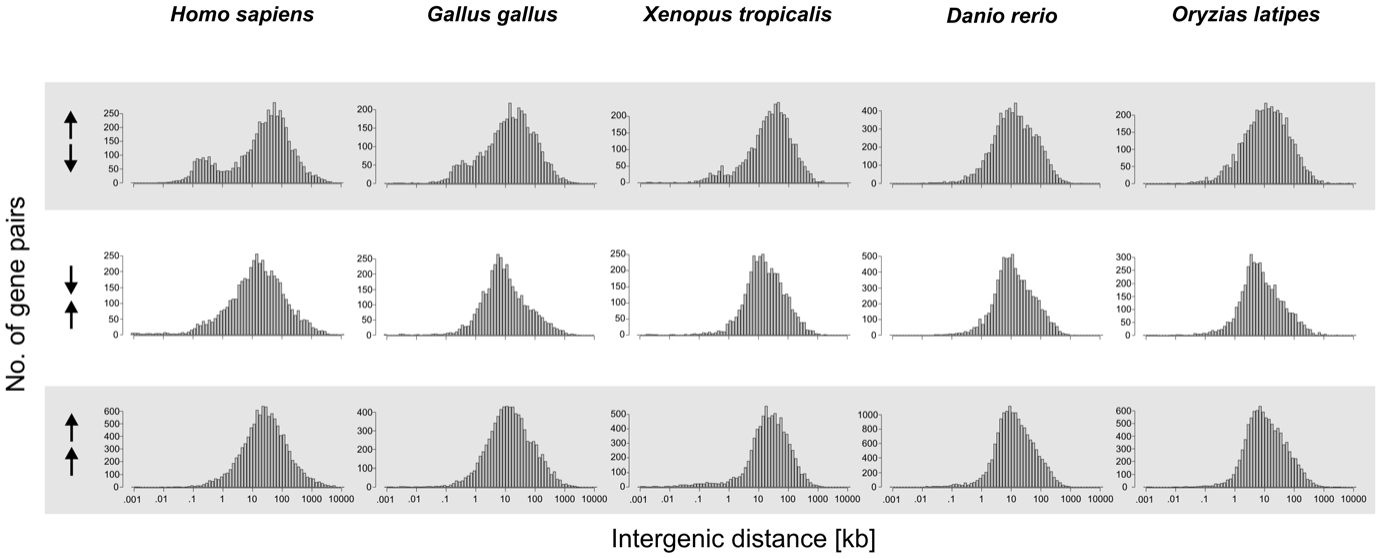
Size distribution of intergenic regions in vertebrates. Distribution of intergenic distances among divergently (← →), convergently (→ ←) and co-directionally (→→) transcribed gene pairs for selected organisms. An enrichment of bidirectional gene pairs is observed in vertebrates (*Gallus gallus*) but not in fishes (*D. rerio* and *O. latipes*) and non-vertebrate animals (Figure S2).

Although data of the type shown in Figure 4 is not able to demonstrate that divergently transcribed gene pairs with a short intergenic distance are conserved during the evolution of metazoa, there are previous reports that such pairs are enriched in the human genome [8], [14], [15], [16]. We therefore examined the distribution of intergenic region sizes in all species considered here (Figures 5 and S2). Consistent with previous reports [8], [30] we noted for the human genome a bimodal distribution of intergenic distances where one of the peaks shows an enrichment of intergenic regions in the size range 100–1000 nt. This is characteristic of pairs of genes that are divergently transcribed, and is not observed for co-directional or convergent pairs (Figure 5). Such an enrichment was also observed in other mammals such as rat and mouse (see also [30]). However, the bimodal distribution was not as marked in birds and in frogs and not at all detectable in the fishes examined here, i.e *Tetraodon nigroviridis*, *Fugu rubripes*, *Gasterosteus aculeatus*, *Oryzias latipes*, and *Danio rerio*. The enrichment of short intergenic regions for divergently transcribed genes therefore seems to have been developed during the evolution of terrestrial vertebrates and is most significant in mammals.

### In pairs of divergently transcribed genes that are evolutionary conserved the genes are likely to be functionally related

For prokaryotes it has been demonstrated that functional associations may be predicted from conserved divergently transcribed genes [29], [31]. Here we wanted to examine if this applies to eukaryotic species. We therefore examined how relative gene orientation and evolutionary conservation of gene pairs are related to functional relationships between the genes as inferred from GO terms. We analyzed genes of Metazoa and Fungi separately as we noted they behaved differently.

**Figure 6:**
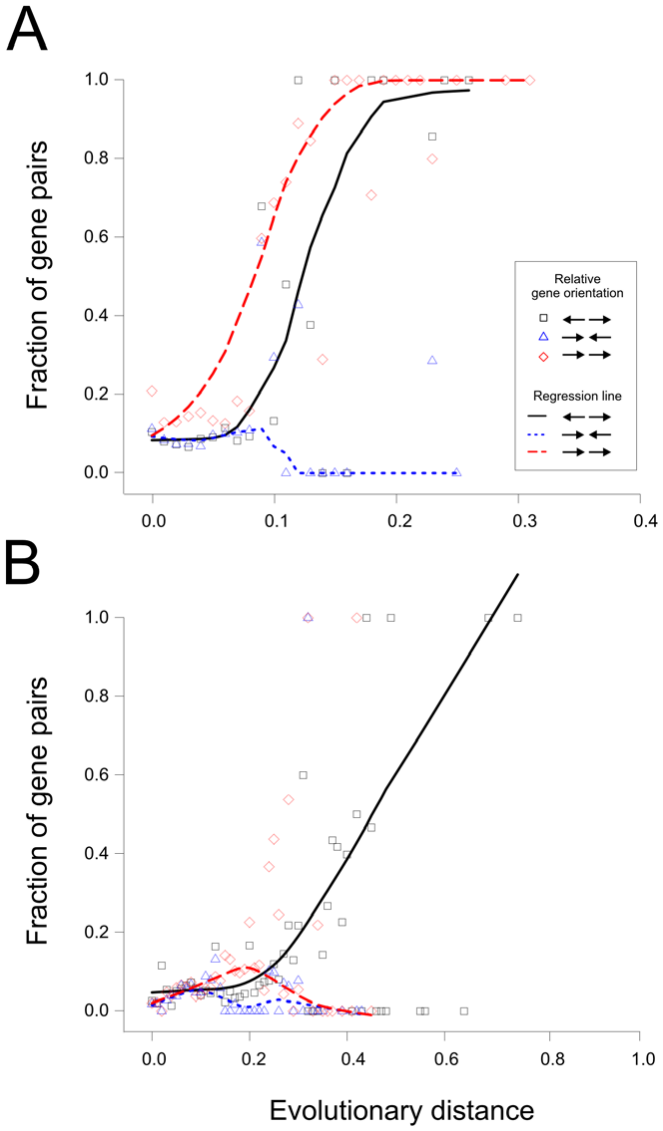
Functional relationship of adjacent genes. Gene pairs of Metazoa (panel A) and Fungi (panel B) are analyzed with respect to evolutionary conservation, relative gene orientation and functional similarity. For a range of evolutionary distances within an interval of 0.01 units the fraction of gene pairs where both genes have a GO similarity score larger than 0.4 [51] were calculated and plotted. For this plot genes were originally clustered with OrthoMCL.

For gene pairs of Metazoa where the genes are divergently or co-directionally transcribed the fraction of gene pairs that are related by GO increases with the measure of evolutionary conservation (Figure 6A, showing results based on OrthoMCL clustering). Thus, as we consider pairs that are strongly conserved during evolution the genes in this pair are likely to be related by function. This does not apply to convergently transcribed genes. For Fungi a similar result is obtained except that here only divergently transcribed genes tend to be functionally related (Figure 6B). This difference between Metazoa and Fungi is presumably because there are many more pairs of genes in Metazoa as compared to Fungi that are the result of gene duplication events. In fact, if all gene pairs in Metazoa where the two genes have the same cluster assignment are removed from the analysis only divergently transcribed genes are related at larger evolutionary distances (data not shown).

The data in Figure 6 indicate that for gene pairs that are evolutionary conserved and that are divergently transcribed, the probability is high that the two genes in the pair are related by function. This is particularly significant in the case of Fungi.

A list of specific pairs ordered according to the measure of evolutionary conservation is in Table 1. The large majority of these pairs involve fungal species. There are examples of gene pairs previously known to be evolutionary conserved, such as H2A/H2B and H3/H4. More interestingly, our results show more examples that were not previously recognized. There are pairs of ribosomal proteins, L21-A/S9-A and S16/L13, as well as pairs of genes with other obvious functional relationships such as two genes involved in pyridoxine biosynthesis (in Saccharomycotina and Pezizomycotina), two iron transport proteins (in Zygomycotina, Basidiomycota, Pezizomycotina), two different mitochondrial heat-chock proteins (in Pezizomycotina), and RNA polymerase subunit RPABC2/Transcription factor IIIA (in Pezizomycotina and Saccharomycotina). Another example is the pair of the DNA repair proteins Rad16 and Rad7 (in Pezizomycotina) that are functionally linked as they are in a complex and a part of the yeast nucleotide excision repair [32].

**Table 1:** Evolutionary conserved gene pairs.

No. Org	No. Gene pairs	Evol. Cons.	GS2 score	GO	Gene 1 description	Gene 2 description	Func. Rel.	Phylum
9	9	1.13	0.80	*	40S ribosomal protein S16	60S ribosomal protein L13	✓	Fc Fm Fs Ft Pr
24	68	0.95	0.63	*	Histone H2B	Histone H2A	✓	Fb Fc Fp Fs Ft Fz M
8	24	0.75	0.84	*	ATP-binding cassette sub-family A	ATP-binding cassette sub-family A	✓	Fc H M V Pr
19	21	0.64	0.00		60S ribosomal protein L21	40S ribosomal protein S9	✓	Fb Fc Fp Fz
12	14	0.60	0.75	*	Probable pyridoxine biosynthesis protein SNZ1	Probable glutamine amidotransferase SNO1	✓	Fp Fs M Pr
17	17	0.56	0.38	*	DNA replication licensing factor MCM2	Protein mlo2		Fp Fs Ft
16	16	0.56	0.00		Putative Xaa-Pro aminopeptidase - Uncharacterized peptidase C22G7.01c	Importin beta-like protein kap111 - Pleiotropic drug resistance regulatory protein 6 - Tryptophanyl-tRNA synthetase, mitochondrial		Fp Fs Ft
17	17	0.55	0.25	*	Eukaryotic initiation factor 4A-III	Pre-mRNA-splicing factor PRP9		Fb Fc Fp
9	9	0.55	1.00	*	Chitin synthase	Chitin synthase	✓	Fp H
4	19	0.54	0.62	*	Histone H2B	Histone H2A	✓	Fs M V
16	21	0.49	0.80	*	Histone H4	Histone H3	✓	Fp Fs Ft
12	12	0.48	0.10	*	Inositol hexakisphosphate and diphosphoinositol-pentakisphosphate kinase	U3 small nucleolar RNA-associated protein 21 - Uncharacterized WD repeat-containing protein C1672.07		Fp Fs Ft
11	11	0.48	0.30	*	Uncharacterized protein C11G11.07 - mRNA transport regulator MTR10	Probable small nuclear ribonucleoprotein E		Fb Fp Fs Ft
15	15	0.47	0.27	*	Pre-mRNA-splicing factor SYF1	Vacuolar proton pump subunit D		Fp Fs
14	14	0.46	0.00	*	60S ribosomal protein L11	Small nuclear ribonucleoprotein-associated protein B		Fb Fp Ft
14	14	0.46	0.22	*	Ribosome biogenesis protein RLP24	Mitochondrial import inner membrane translocase subunit TIM14		Fp Fs
17	17	0.46	0.13	*	ATP-dependent rRNA helicase RRP3	Brix domain-containing protein C1B9.03c - Ribosome biogenesis protein SSF1		Fp Fs
13	13	0.45	0.00		Uncharacterized protein	Uncharacterized protein		Fp Fs
14	14	0.45	0.43	*	U3 small nucleolar RNA-associated protein 17	DNA-directed RNA polymerases I, II, and III subunit RPABC5		Fp Fs Ft
2	3	0.45	0.12	*	Protein kinase gsk3	Guanosine-diphosphatase		Fm Fz
12	16	0.44	0.80	*	Iron transport multicopper oxidase FET precursor	Iron transporter FTH1 - Plasma membrane iron permease	✓	Fb Fc Fp Ft Fz
12	17	0.43	0.09	*	Alpha-glucosidase	Alpha-glucosides permease MPH2/3		Fb Fp Fs
7	7	0.43	0.62	*	Homogentisate 1,2-dioxygenase	Fumarylacetoacetase		Fp H
15	15	0.42	0.60	*	Palmitoyltransferase ERF2	Uncharacterized protein C3H7.08c		Fp Ft
15	15	0.42	0.47	*	Protein CASP	Vacuolar protein sorting-associated protein 3		Fp Ft
15	15	0.42	0.45	*	Eukaryotic translation initiation factor 5A-1,2	Vacuolar protein sorting-associated protein 52		Fp Ft
15	15	0.42	0.11	*	Vacuolar protein-sorting-associated protein 24	Protein wos2		Fp Ft
15	15	0.42	0.00		Uncharacterized WD repeat-containing protein	RNA processing protein efg1		Fp Ft
15	15	0.42	0.00		Regulator of ribosome biosynthesis	37S ribosomal protein S23, mitochondrial		Fp Ft
4	4	0.41	0.19	*	Pre-mRNA-splicing factor CWC24 (Complexed with CEF1 protein 24)	Co-chaperone protein HscB, mitochondrial precursor		Fs V
14	14	0.41	0.09	*	Actin-related protein 2/3 complex subunit 4	Ubiquitin carboxyl-terminal hydrolase 6		Fp Ft
12	12	0.41	0.30	*	ATP-dependent RNA helicase DBP5	Uncharacterized protein C12C2.05c		Fp Ft
14	14	0.40	0.91	*	DNA repair protein RAD16	DNA repair protein RAD7	✓	Fp Ft
13	13	0.40	0.35	*	Calcineurin subunit B	Enhancer of polycomb-like protein 1		Fp Ft
15	15	0.40	0.24	*	DNA-directed RNA polymerase III subunit RPC3	Cytochrome b-c1 complex subunit 2, mitochondrial precursor		Fp Fs
14	14	0.40	0.69	*	DNA-directed RNA polymerases I, II, and III subunit RPABC2	Transcription factor IIIA	✓	Fp Fs
11	11	0.40	0.00	*	Cullin-3	Uncharacterized protein C24H6.02c		Fp Ft
13	13	0.40	0.51	*	Serine/threonine-protein kinase chk1	Ubiquitin-conjugating enzyme E2-20 kDa		Fp Ft
13	13	0.40	0.24	*	Eukaryotic peptide chain release factor GTP-binding subunit	Ran-specific GTPase-activating protein 30		Fp Ft
10	10	0.40	0.00		Protein pdh1 precursor - Uncharacterized membrane protein YOL107W	Uncharacterized WD repeat-containing protein C1235.09		Fp Ft
12	12	0.39	0.53	*	Elongation of fatty acids protein 2	Cytochrome c oxidase polypeptide VI, mitochondrial precursor		Fp Ft
13	13	0.39	0.00		Probable 60S ribosomal protein L28e	UPF0357 protein C1687.07 precursor		Fp Ft
10	10	0.39	0.27	*	Geranylgeranyl transferase type-2 subunit alpha	Meiosis-specific APC/C activator protein AMA1		Fp Ft
14	14	0.39	0.71	*	60 kDa heat shock protein, mitochondrial precursor	10 kDa heat shock protein, mitochondrial precursor	✓	Fb Fc M
8	12	0.39	0.00		Beta-1,3-glucan-binding protein precursor			Fb Fp Fs
6	6	0.39	0.00		40S ribosomal protein S15	60S acidic ribosomal protein P2-beta	✓	Fb Fs Ft
13	16	0.38	1.00	*	3-oxoacyl-(acyl-carrier-protein) synthase	S-acyl fatty acid synthase thioesterase	✓	Fb Fp
2	2	0.38	0.00		Vesicle associated membrane protein	DNA excision repair protein ERCC-1		V Pr
12	12	0.38	0.42	*	Histone deacetylase	Chromatin modification-related protein YNG2		Fp Ft
11	11	0.38	0.23	*	Biotin ligase	Mitochondrial genome maintenance protein MGM101, mitochondrial precursor		Fp Ft
3	4	0.38	0.65	*	ATP synthase subunit beta, mitochondrial precursor	ATP synthase subunit delta, mitochondrial precursor	✓	Fc Fz H

Gene pairs are ordered according to evolutionary conservation. First column shows the number of species where a particular gene pair is present. Second column shows the total count of the gene pair in all species where it occurs. A star (*) indicates that both genes in a pair have a GO annotation. Functional relationships were inferred by mining of literature. Fp, Pezizomycotina; Fs, Saccharomycotina; Ft, Thaphrinomycotina; Fb, Basidiomycota; Fc, Chytridiomycota; Fm, Microsporidia; M, Mammals; V, Viridiplantae; Pr, Protozoa; H, Heterokonta.

A particularly interesting example of conserved gene order is the pair of genes encoding the ribosomal proteins S16 and L13 (Table 1). This gene pair is found in the fungal species *Encephalitozoon cuniculi*, *Batrachochytrium dendrobatidis*, *S. cerevisiae*, *Lodderomyces elongisporus* and *Schizosaccharomyces pombe*, as well as in the apicomplexa *Cryptosporidium parvum*, *Plasmodium falciparum*, *Theileria parva* and *Toxoplasma gondii*. The significance of this gene pair is discussed further below.

Also metazoan gene pairs that are divergently transcribed and that are evolutionary conserved tend to be enriched for gene pairs with common function as inferred from GO (Table S4), although our measure of evolutionary conservation is not as large as that for Fungi (compare Figures 5A and B). Examples of conserved gene pairs are the genes encoding the heat shock proteins Hsp10 and Hsp60 and the histone H2A/H2B genes. (Table S4).

### Potential bidirectional promoters in the human genome

Pairs of divergently transcribed genes with a short intergenic distance are enriched in the human genome. Such gene pairs are assumed to have bidirectional promoters. They are identified in Table S4, but to focus on the human genome we specifically examined human gene pairs of this kind that are evolutionary conserved. We identified a total of 5,855 divergently transcribed gene pairs in the human genome. Out of these, 924 gene pairs were separated by less than 1000 base pairs and were found in at least one more species (Table S5). Such pairs are shown in the Table S5 and are ordered according to evolutionary conservation. Analysis of this list shows that only about 6% of the gene pairs have previously been shown to be regulated by bidirectional promoters and only 8.5% of the total were considered to be functionally related based on the GO score. However, examining the list of pairs being most strongly conserved as shown in Table 2, we noted that six of these pairs had previously been described as having bidirectional promoters. In Table S5 are highlighted more examples of such promoters that were previously described. Therefore, the top candidates in Table 2 as well as those of the Table S5 may contain more examples of such pairs (see also under “Discussion”).

**Table 2:** Conserved pairs of divergently transcribed genes from human.

Gene 1	Gene 2	Evolutionary conservation	Intergenic distance	GS2 score	Gene 1 description	Gene 2 description	Functional relationship	References
HIST1H2AJ	HIST1H2BM	0.54	304	0.62	Histone H2A type 1-J	Histone H2B type 1-M	✓	[52]
HSPD1	HSPE1	0.39	49	0.71	60 kDa heat shock protein, mitochondrial precursor	10 kDa heat shock protein, mitochondrial	✓	[53]
IMMP1L	ELP4	0.28	128	0.34	Mitochondrial inner membrane protease subunit 1	Elongator complex protein 4		
PPAT	PAICS	0.43	70	0.38	Amidophosphoribos yltransferase precursor	Multifunctional protein ADE2	✓	[54], [55]
GBA2	RGP1	0.16	115	0	Non-lysosomal glucosylceramidase	Retrograde Golgi transport protein RGP1 homolog		
COL4A1	COL4A2	0.15	118	0.76	Collagen alpha-1(IV) chain precursor (Arresten)	Collagen alpha-2(IV) chain precursor	✓	[18]
DUOX2	DUOXA2	0.14	<0	0.17	Dual oxidase 2	Dual oxidase maturation factor 2	✓	[56]
DUOXA1	DUOX1	0.14	135	0.17	Dual oxidase maturation factor 1	Dual oxidase 1	✓	[56]
RTN4IP1	QRSL1	0.13	80	0.75	Reticulon-4-interacting protein 1, mitochondrial precursor	Glutamyl-tRNA(Gln) amidotransferase subunit A homolog		
LRBA	MARB21L2	0.13	<0	0.14	Lipopolysaccharide-responsive and beige-like anchor protein	Protein mab-21-like 2		

Ten most conserved human bidirectional gene pairs where only those with an intergenic distance less than 1000 base pares are included. Functional relationships were inferred by mining of literature. For a more comprehensive list of gene pairs see Table S4.

Further examination of the gene pairs in Table 2 reveals that five are clearly related by function, i.e. the 60 kDa/10 kDa heat shock proteins, histones H2A/H2B, collagen type IV alpha 1 and 2, and the DUOX2/DUOXA2 and DUOXA1/DUOX1 pairs. Of these, the heat shock proteins as well as the dual oxidase/dual oxidase maturation proteins are pairs where the proteins are non-homologous. In conclusion, it would therefore seem that the gene pairs in Table 2 are highly likely to involve bidirectional promoters and to have genes that are functionally related.

## Discussion

We have compiled information about the location of protein-coding genes in 64 different eukaryotic species. Orthologue and homologue relationships were identified with two different methods, OrthoMCL and Pfam classification. The aim of OrthoMCL is to generate clusters where the members of each cluster are orthologous, although the clustering is somewhat ambiguous as it is dependent on parameters that are supplied to the program and is also dependent on the actual set of protein sequences used. The situation with the classification based on Pfam is different as a Pfam family may contain both orthologues and paralogues. As a result, the classification according to Pfam is expected to generate fewer clusters as compared to OrthoMCL. The fact that we reduced the complexity of Pfam architectures also contributed to this effect. Indeed, only 17,171 Pfam groups were identified as compared to 71,219 clusters with OrthoMCL. The fact that OrthoMCL gave rise to a larger number of clusters also had the effect that a smaller evolutionary distance between gene pairs was observed.

In order to compare gene order in different species a classification based on orthology would be ideal and therefore, OrthoMCL would be more relevant than the classification based on Pfam. On the other hand, potential orthologues are identified using BLAST in the OrthoMCL method and in cases where the orthology relationship is not easily revealed using BLAST, Pfam may be more efficient. An advantage with Pfam classification is also that there are very few false positives.

Among gene pairs that are conserved during evolution the divergent and co-directional gene pairs are much more common than convergent pairs (Figure 3), consistent with previous studies of bacterial genes [29]. In the case of bacteria, co-directional gene pairs are common because of polycistronic operons. In eukaryotes co-directional pairs should be less frequent than in bacteria. This is indeed observed in Figure 3A. On the other hand, the results based on Pfam clustering (Figure 3B) show that the co-directional pairs are more common in a long range of evolutionary distances as compared to the results based on the OrthoMCL clustering. This is possibly reflecting the fact that there are many co-directional gene pairs that are the result of gene duplication and where the two genes are paralogues. As Pfam classification will typically not distinguish between paralogues, the Pfam based grouping will in many cases erroneously identify gene pairs in different species as being the same.

We have shown that for pairs of genes that are evolutionary conserved and that are divergently transcribed the genes involved are likely to be functionally related. One of the most strongly conserved gene pairs is that of genes encoding the ribosomal proteins S16 and L13. This gene pair is found in fungal species as well as in apicomplexa. It could have been formed by way of convergent evolution at many instances during evolution. Alternatively, it represents a gene pair present early in evolution that was lost in many phyla. In this regard it is of interest to note that this pair is present also in bacteria and eubacteria, where the S16 homologue is referred to as S9. In a vast majority of eubacteria (for example *Escherichia coli* [33]) and archaea (for example *Haloarcula marismortui* [34]) the S9 and L13 genes are positioned next to each other as part of the same operon. In *Methanobacterium thermoautotrophicum* these two proteins have been fused [35]. Therefore, in all kingdoms of life we see examples where these two proteins are related through gene organisation. It seems highly likely that the genes of the eukaryotic pair are related in terms of transcription. A balanced production of these two proteins might be critical for ribosome assembly or function. There is no obvious relationship of the two proteins as to their positions in the ribosome. In the bacterial ribosome the two proteins are both located distantly from the interface between the two ribosomal subunits and on opposite sides of the 70S particle.

Yet another evolutionary conserved pair of ribosomal protein genes are the L21 and S9 proteins. This pair is found in as diverse fungal branches as Pezizomycotina, Basidiomycota, Zygomycota and Chytridiomycota. Whereas the S16 and L13 proteins are transcriptionally related in eubacteria and archaea this does not seem to be the case for the L21 and S9 proteins.

Also among highly conserved gene pairs in metazoa, we have noted en enrichment for pairs where the two genes are functionally related. Examples are the Hsp10 and Hsp60 proteins (Tables 2, S4 and S5). Thus, both in metazoa and fungi there is a positive correlation between evolutionary conservation and functional relationship. This observation indicates that among highly conserved gene pairs, functional relationships may be predicted in cases where this is not obvious already from available annotation. On the other hand, such predictions do not seem as reliable and extensive as in the case of bacteria [29], [31].

The results of this work also allow identification of potential bidirectional promoters. We observed that in a list of human genes that are divergently transcribed and that are characterized by a short intergenic distance, a number of gene pairs are found that previously have been characterized as having bidirectional promoters (Table 2, Table S5). This relationship is particularly strong when considering genes that are related by GO. We are therefore able to predict gene pairs regulated by bidirectional promoters. Examples are three olfactory receptor gene pairs (O51B2/O51B6, O51I1/O51I2, OR8K5/OR5J2), two subunits of the ligand-gated ion channel (GABRB3/GABRA5) and two heat shock proteins (HSPA1L/HSPA1A) (Table 2, Table S5). At the same time, a lack of functional similarity does not exclude the possibility that a given gene pair is regulated by a bidirectional promoter. One example is the gene pair PLEKHJ1/SF3A2 (a guanine nucleotide releasing protein/spliceosome-associated protein 62) where the advantage of sharing a bidirectional promoter has not been fully understood [36]. More examples are found in Table S5. It is important to note that there are also divergently transcribed genes that have a bidirectional promoter and that are separated by more than 1,000 bps, e.g. FANCA/SPIRE2[9], BCRA2/DR731263[9], PREPL-C2ORF34 [37], CYP1A1/CYP1A2 [38] (23,3037) and FANCF/GAS2[9]. It must finally be noted that only protein-coding genes were considered in this investigation. There are previously known pairs of divergently transcribed genes that involve non-coding RNA genes that have shown similar expression profiles, suggesting a transcriptional regulation mediated by a bidirectional promoter [21], [39].

In summary, we have examined parameters related to gene order conservation in eukaryotes and have found that evolution of gene pairs is constrained in a number of situations. In metazoa, co-directional gene pairs tend to be conserved. A possible explanation is that such pairs are related in terms of transcriptional control. In both metazoa and fungi, divergently transcribed pairs of genes, often with a short intergenic distance, are conserved. For many such pairs the transcription of the two genes are likely to be related, for instance because of overlapping promoter elements. As a consequence, the genes in the pair cannot easily be separated by a recombination event. In addition, it is possible that the transcriptional relationship in such a pair is beneficial and that it is kept during evolution for this reason.

Certain gene pair categories are also likely to be related in terms of function. This applies mainly to evolutionary conserved, divergently transcribed genes in fungi and metazoa and co-directionally transcribed genes in metazoa. A plausible explanation to this functional relationship is that during evolution, adjacent genes that are functionally related more easily develop a transcriptional relationship. For instance, this relationship could ensure that the two proteins are produced in comparable amounts, or that the proteins are anti-regulated. A major conclusion from this work is therefore that studies of gene order conservation aid in identifying genes that are related in terms of transcriptional control.

## Materials and Methods

### Sources of genomic and protein sequences

Genomic and protein sequences were obtained from NCBI (http://www.ncbi.nlm.nih.gov/entrez/; ftp://ncbi.nih.gov/genomes), SWISSPROT (http://www.uniprot.org/), ENSEMBL (http://www.ensembl.org), TIGR (ftp://ftp.tigr.org/pub/data/), the U.S. Department of Energy Joint Genome Institute (http://www.jgi.doe.gov), the Sanger Institute (http://www.sanger.ac.uk), the HGSC at Baylor College (http://www.hgsc.bcm.tmc.edu/projects/), BROAD Institute (http://www.broad.mit.edu/annotation/fgi/) as well as specific genome project databases: SGD (http://www.yeastgenome.org/), PlasmoDB (http://www.plasmodb.org), ToxoDB (http://www.toxodb.org/toxo/home.jsp), DictyBase (http://dictybase.org/), the *Cyanidioschyzon merolae* Genome Project (http://merolae.biol.s.u-tokyo.ac.jp) and the Antonospora locustae DB (http://gmod.mbl.edu/perl/site/antonospora01?page=download). More details on database versions are in Table S1.

### Compilation of gene order information

Information about protein encoding genes, such as genomic location, strand information and protein sequence was compiled from a variety of databases sources (Table S1). For the majority of genomes considered we made use of existing annotation. When nucleotide positions of protein encoding genes were not directly available from the different web repositories, a protocol was used in order to map the protein sequence to its corresponding nucleotide location. Initially, a TBLASTX [40] search was performed using a given protein as query and the specific organism genomic sequence as database. HSPs with at least 80% of identity were considered for further analysis. In cases where the percentage of identity was 100%, the positions of these hits were considered reliable. GeneWise[41] was used to predict the gene structure of the remaining sequences. The start and end sites of a gene in this work are defined as the 5′ and 3′ ends of the coding sequence, respectively.

Existing gene information as well as in house annotation resulted in a total of 1,113,045 proteins. In this material overlapping protein sequences were present as a result of alternative splicing. We removed this type of redundancy such that whenever overlapping annotated coding sequences were present on the same strand and chromosome/contig/supercontig only the splice variant corresponding to the longest protein sequence was kept. After such removal of alternative splicing products a total of 823,840 proteins/genes remained. For this final set of genes, intergenic distances were calculated. Information about pairs of adjacent genes was compiled and classified according to their relative orientation, i.e convergent, divergent or co-directional. For more details see Tables S1, S2, and S3.

For the more computationally demanding operations in this work, we used computing resources at the Chalmers Centre for Computational Science and Engineering, Chalmers University of Technology/University of Gothenburg, Sweden.

### Identification of orthologues using OrthoMCL

Proteins were clustered using OrthoMCL v2.0 [25], a method for constructing orthologous groups across multiple eukaryotic taxa that uses a Markov Cluster algorithm to group (putative) orthologues and paralogues. Poor quality sequences (19 proteins) were first filtered out based on length (less than 10 amino acids) and percent of stop codons (>20%). All-vs-all BLAST searches were then performed and the results of those searches were used as input to OrthoMCL. Cutoffs for the percent match and e-value were set to 10 and 1e-5, respectively.

### Grouping of protein sequences based on Pfam architectures

In order to assign known functional domains to each one of the proteins in the dataset, searches using hmmpfam of the HMMER 2.3.2 package [42] were performed with the Pfam database (version 23, August 2008) [26] as the library of HMMs. The trusted cutoff (the lower score for sequences belonging to the full alignment for a given Pfam family) was used as inclusion threshold. Pfam domains were identified in 458,597 proteins (56% of the total number of proteins). These proteins were then classified on the basis of Pfam architecture (i.e. an order of Pfam domains present in a given protein, such as the two domain architecture ‘DEAD∼Helicase_C’). This classification is based on the assumption that two proteins with the same architecture have related functions. We reduced the complexity of architectures so that whenever a protein has domain repeats, only one copy was retained. For instance, a protein with Pfam architecture “PPR∼PPR∼PPR” was reduced to “PPR”. This procedure assumes that two proteins with different numbers of domain repeats are functionally related. The total number of Pfam architectures, before and after reduction as described above, was 26,666 and 17,171, respectively.

A fraction of OrthoMCL clusters and Pfam architectures, 32% and 45%, respectively, had sequences from a single organism only. Such clusters and architectures were excluded from further analysis since the purpose of this study was to investigate the conservation of gene order across species.

### Phylogenetic tree and evolutionary distances

With the aim of estimating the evolutionary distance associated with a set of species a phylogenetic tree was constructed. The topology of the tree was first set manually using available phylogenetic information [43], [44], [45], [46], [47]. Branch lengths of the tree were then determined on the basis of a multiple alignment of four proteins, α-tubulin, β-tubulin, actin and the elongation factor 1-alpha (EF-1α) [43]. The amino acid sequences of these proteins were retrieved from all the organisms used in this study (Table S1, Figure 1). The sequences from each species were then concatenated and a multiple alignment was produced using ClustalW 1.83 [48]. From the alignment gap columns were removed using GapStreeze (http://hiv-web.lanl.gov/content/hiv-db/GAPSTREEZE/gap.html). The alignment was then used to construct a distance matrix using PROTDIST and branch lengths of the above tree were then estimated using PROTPARS. PROTDIST and PROTPARS are from the PHYLIP package [49].

A measure of evolutionary conservation for each gene pair present in more than one species was calculated as the sum of branch lengths for the species involved. The sum of branch lengths of a set of nodes in the phylogenetic tree (Figure 1) was calculated using a perl script using as input a list of the nodes as well as the full tree.

### Gene Ontology term assignments

A Gene Ontology (GO) database of September 18, 2009 was downloaded from the Gene Ontology website (http://www.geneontology.org/). In order to annotate our protein set with respect to GO terms, BLASTP searches were performed against a reference protein dataset as database. This dataset was a subset of proteins from the Uniprot [50] Knowledgebase with gene ontology annotation (233,689 proteins). Each protein was assigned the GO term(s) that corresponded to the best hit in this search, assuming that the E-value was less than 1e-20. In cases where no term could be assigned to a protein using this method, we used GO terms that were representative of the OrthoMCL cluster or Pfam group to which it belonged. With this procedure approximately 41% of the dataset (338,964 proteins) received a GO term. When assigning GO terms to proteins the common GO terms “cytosol”, “nucleolus”, “cytoplasm”, “protein binding” and “nucleus” were removed from all analysis, since these terms are not expected to be informative in the context of this work.

GO terms were assigned not only to individual proteins but also to the OrthoMCL clusters. This was done by combining GO terms obtained from individual proteins in each cluster. A total of 27,031 OrthoMCL clusters were assigned a GO term using this method. In addition, GO terms were assigned to Pfam architectures by making use of GO terms associated with Pfam entries according to the Pfam database. For a multi-domain protein, GO terms were combined from all domains in that protein. A total of 12,052 architectures received a GO classification using this method.

To examine the functional similarity between adjacent genes, we used a GO-based similarity based on the GS^2^ measure [51]. This method obtains a score for the similarity between two sets of genes. In our case, we are comparing only two genes, i.e each set of genes has only one gene. According to Ruths et al [51] the similarity measure is 0.4 for a completely random sets of genes, and we therefore used this as a threshold value.

## Supporting Information

Figure S1 Relative orientation of gene pairs. Selected organisms are shown to illustrate cases where the three relative orientations of transcription (divergent (←→), convergent (→←) and co-directional (→→) are randomly distributed (top) and organisms where such distribution seems less random (bottom).
(0.02 MB PDF)

Figure S2 Distribution of intergenic distances. For all 64 species analyzed in this work the distribution of intergenic distances is shown for all three possible relative gene orientations. The x axis represents intergenic distance, where “1” is 1 kbases.
(0.25 MB PDF)

Table S1 Sources of genomic sequences, statistics on proteins, relative gene orientation, and clustering using OrthoMCL and Pfam.
(0.05 MB PDF)

Table S2 Pairs of adjacent genes. Gene pairs are grouped according to relative gene orientation and according the basis of classification (OrthoMCL or Pfam). To each gene pair is attributed information about evolutionary conservation, score based on GO similarity, GO terms, functional description of proteins, and the species where the pair was identified. Only gene pairs with evolutionary conservation larger than 0.3 are shown.
(8.28 MB XLS)

Table S3 Intergenic distances characteristic of different species.
(0.01 MB PDF)

Table S4 Pairs of adjacent genes in Metazoa. Genes were clustered using OrthoMCL and gene pairs are grouped according to relative gene orientation. To each gene pair is attributed information about evolutionary conservation, intergenic distance, score based on GO similarity, GO terms, functional description of proteins, and the species where the pair was identified.
(9.08 MB XLS)

Table S5 Potential bidirectional promoters. Pairs of divergently transcribed genes with an intergenic distance less than 1000 nt that occur in human and in at least one more species are shown. To each gene pair is attributed information about evolutionary conservation, score based on GO similarity, GO terms, functional description of proteins, intergenic distance and, in cases where applicable, literature references describing the pair.
(0.48 MB XLS)
