# Supplementary figures and images for: Analysis of Gene Order Conservation in Eukaryotes Identifies Transcriptionally and Functionally Linked Genes
Source: PLoS One. 2010 May 14;5(5):e10654. doi: 10.1371/journal.pone.0010654 (PMC2871058; doi:10.1371/journal.pone.0010654)

Figure S1: Relative orientation of gene pairs

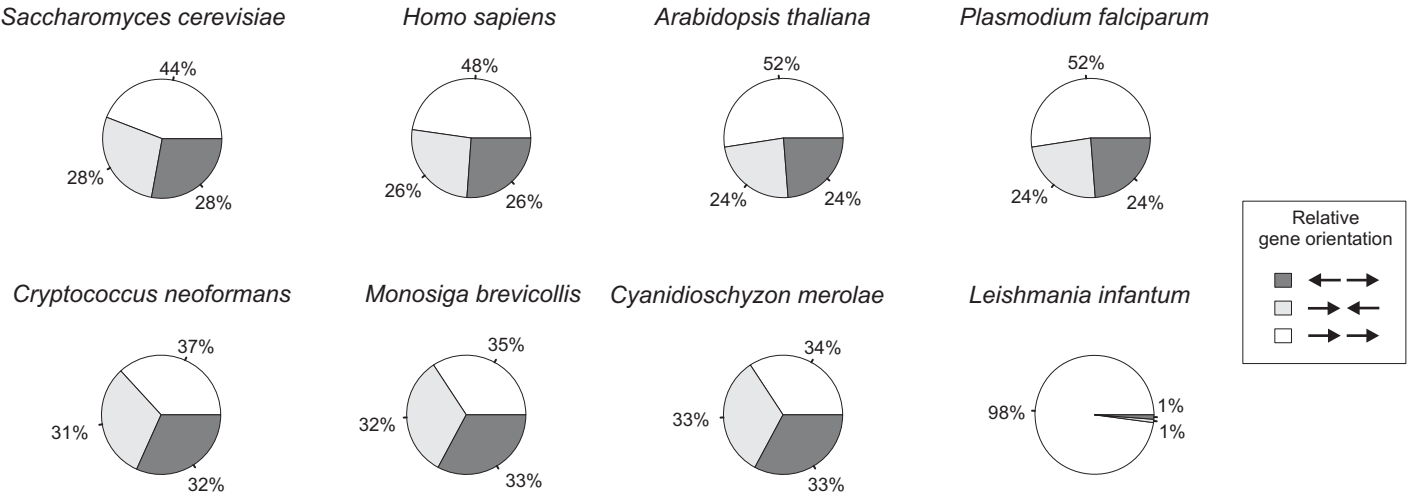

Supplement: Figure S1 — Relative orientation of gene pairs. Selected organisms are shown to illustrate cases where the three relative orientations of transcription (divergent (←→), convergent (→←) and co-directional (→→) are randomly distributed (top) and organisms where such distribution seems less random (bottom). (0.02 MB PDF) [file pone.0010654.s001.pdf]
