# Supplementary material for: Analysis of Gene Order Conservation in Eukaryotes Identifies Transcriptionally and Functionally Linked Genes
Source: PLoS One. 2010 May 14;5(5):e10654. doi: 10.1371/journal.pone.0010654 (PMC2871058; doi:10.1371/journal.pone.0010654)
Supplement: Figure S2 — Distribution of intergenic distances. For all 64 species analyzed in this work the distribution of intergenic distances is shown for all three possible relative gene orientations. The x axis represents intergenic distance, where “1” is 1 kbases. (0.25 MB PDF) [file pone.0010654.s002.pdf]

Figure S2: Distribution of intergenic distances

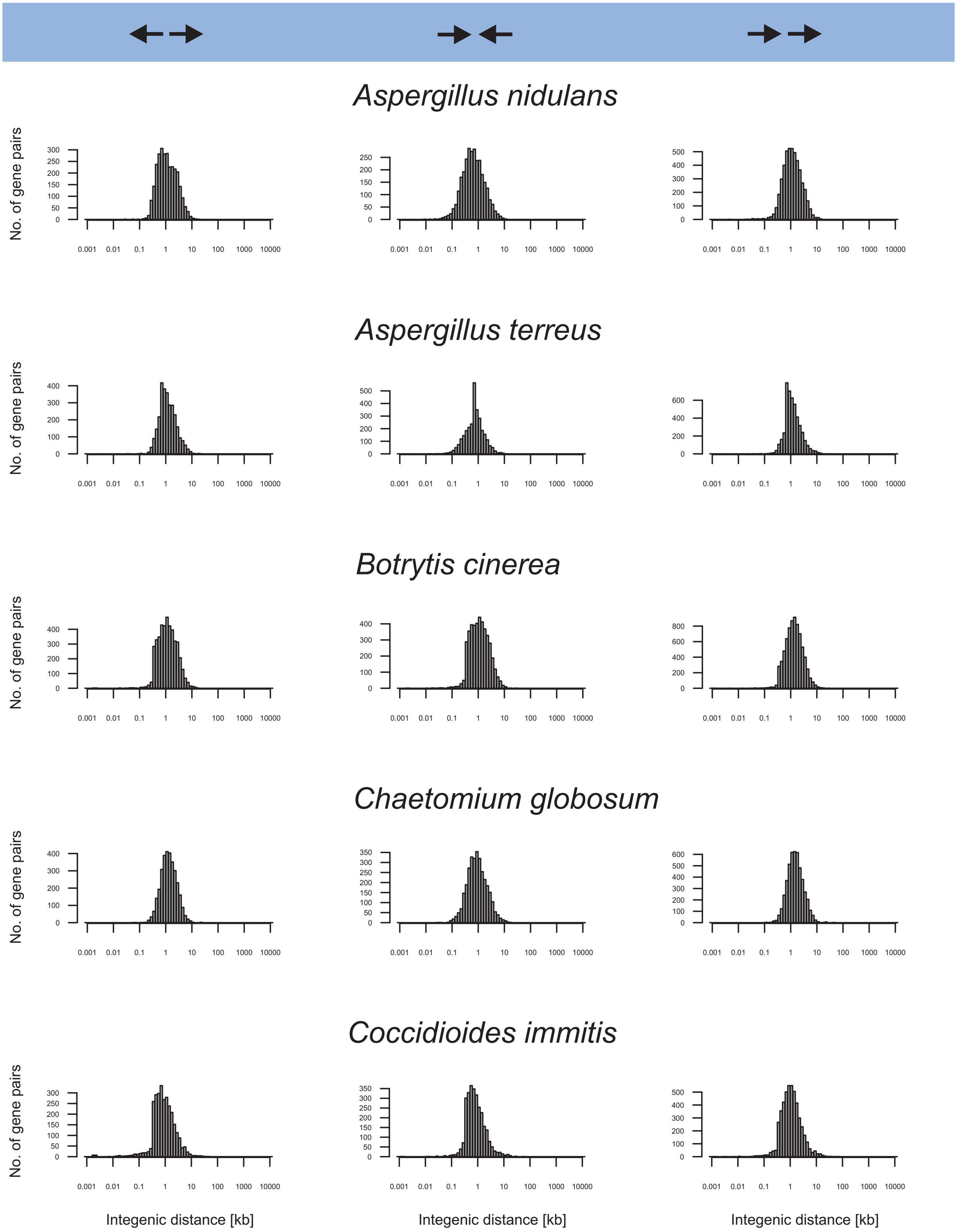

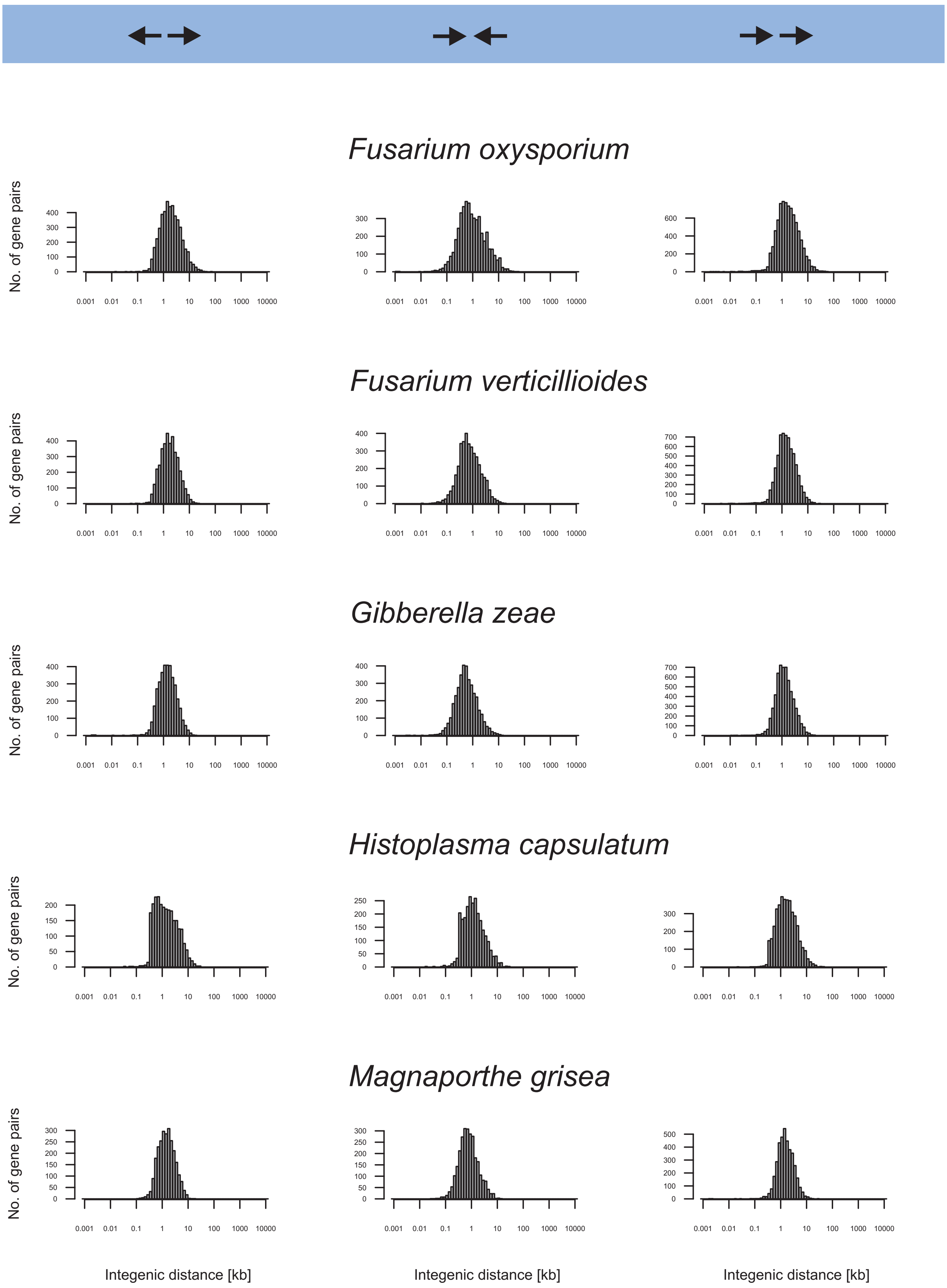

Fusarium oxysporium
Fusarium verticillioides
Gibberella zeae
Histoplasma capsulatum
Magnaporthe grisea
No. of gene pairs
0.001 0.01 0.1 1 10 100 1000 10000
Integenic distance [kb]
Integenic distance [kb]
Integenic distance [kb]

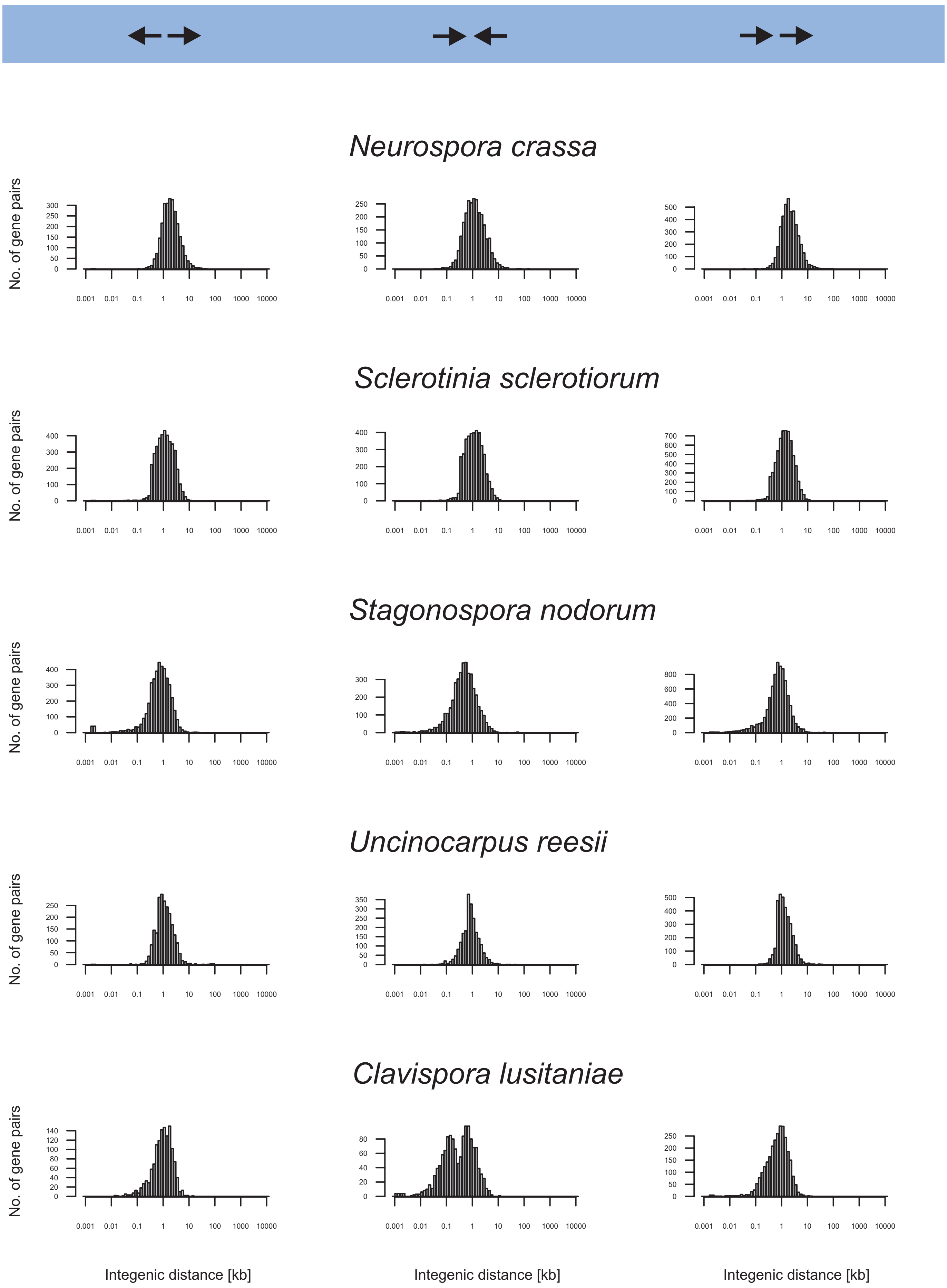
Neurospora crassa
Sclerotinia sclerotiorum
Stagonospora nodorum
Uncinocarpus reesii
Clavispora lusitaniae
No. of gene pairs
0.001 0.01 0.1 1 10 100 1000 10000
Integenic distance [kb]

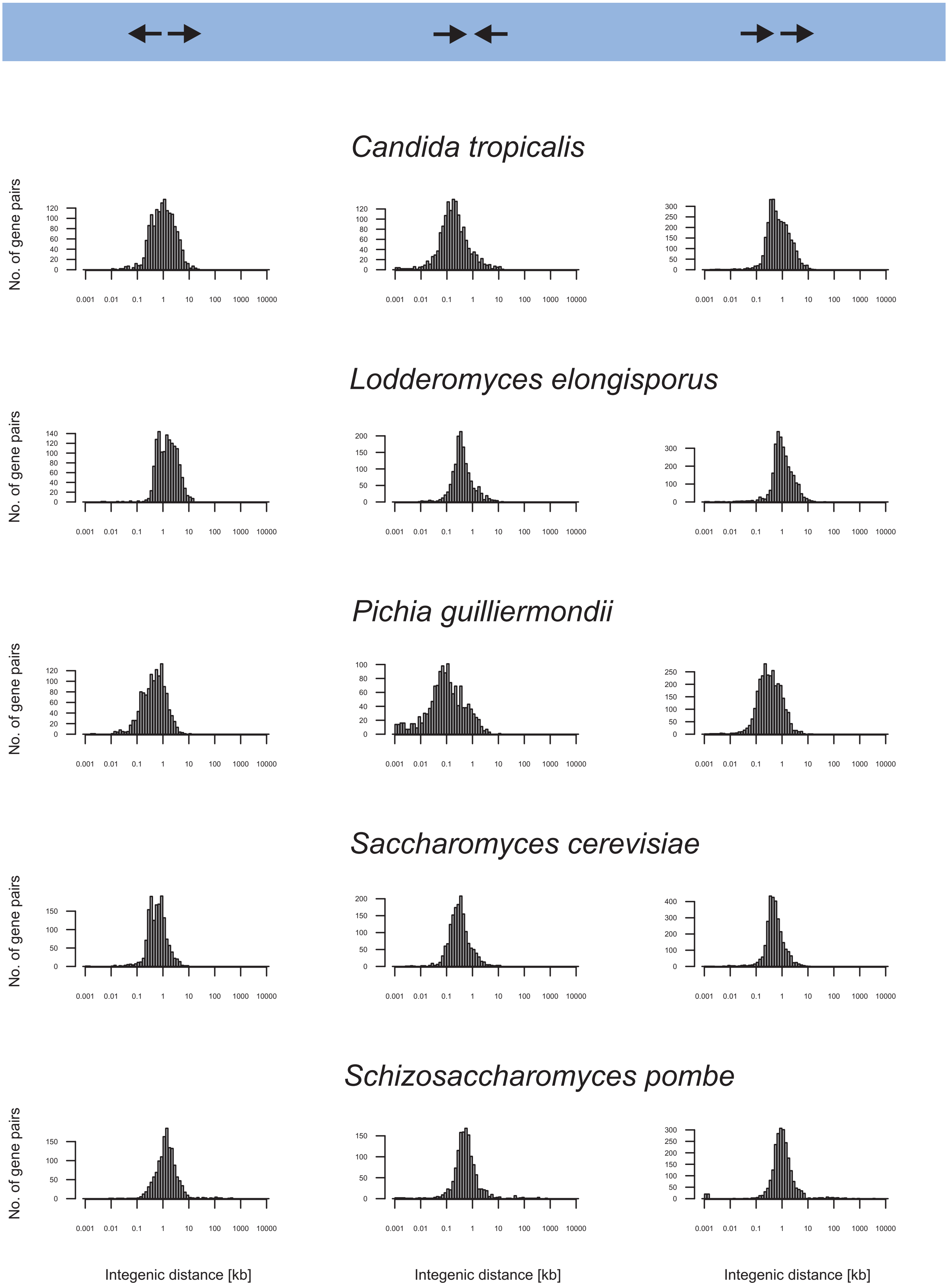
Candida tropicalis
Lodderomyces elongisporus
Pichia guilliermondii
Saccharomyces cerevisiae
Schizosaccharomyces pombe
No. of gene pairs
0.001 0.01 0.1 1 10 100 1000 10000
Integenic distance [kb]
Integenic distance [kb]
Integenic distance [kb]

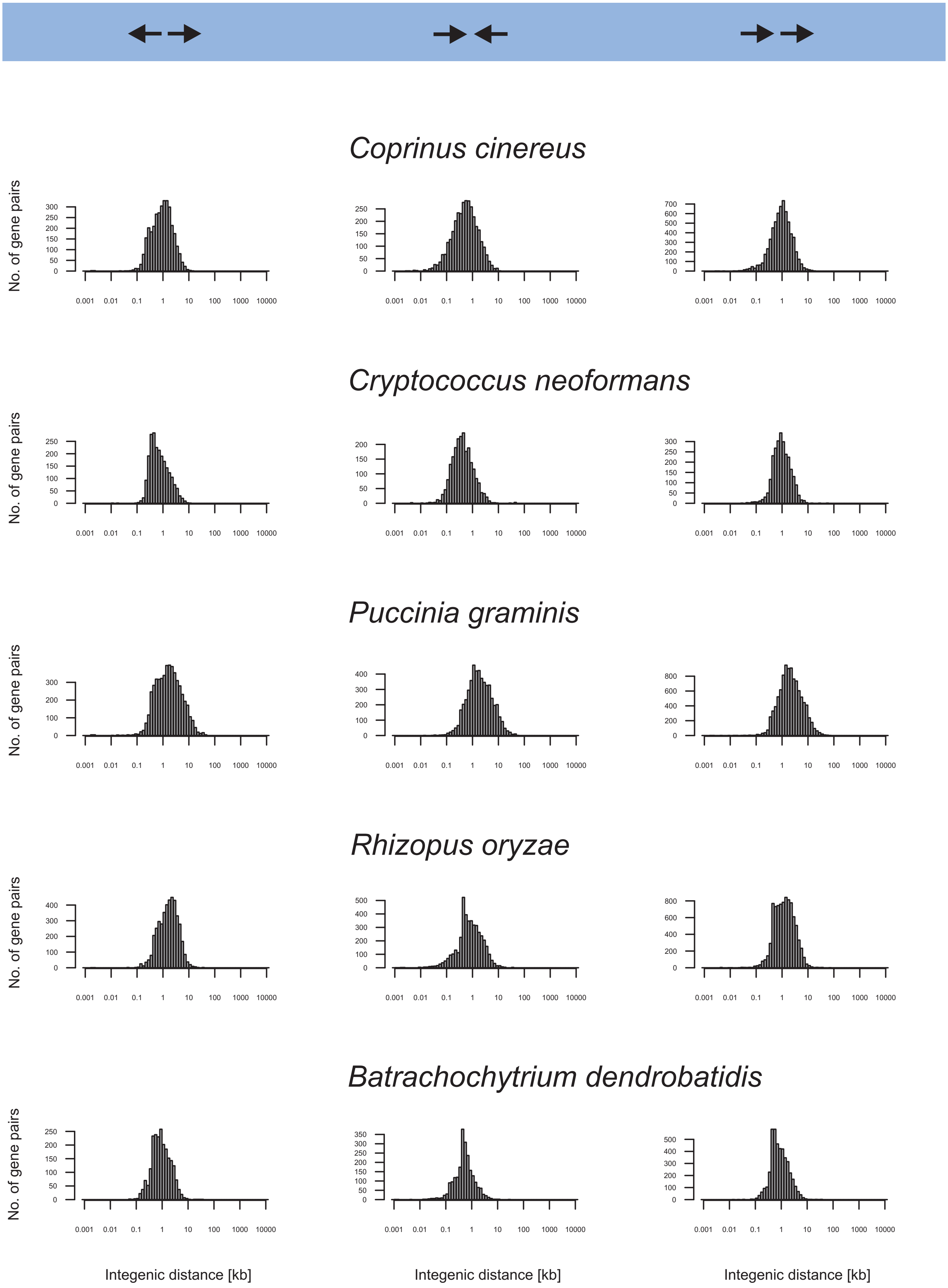

Coprinus cinereus
Cryptococcus neoformans
Puccinia graminis
Rhizopus oryzae
Batrachochytrium dendrobatidis
No. of gene pairs
0.001 0.01 0.1 1 10 100 1000 10000
Integenic distance [kb]

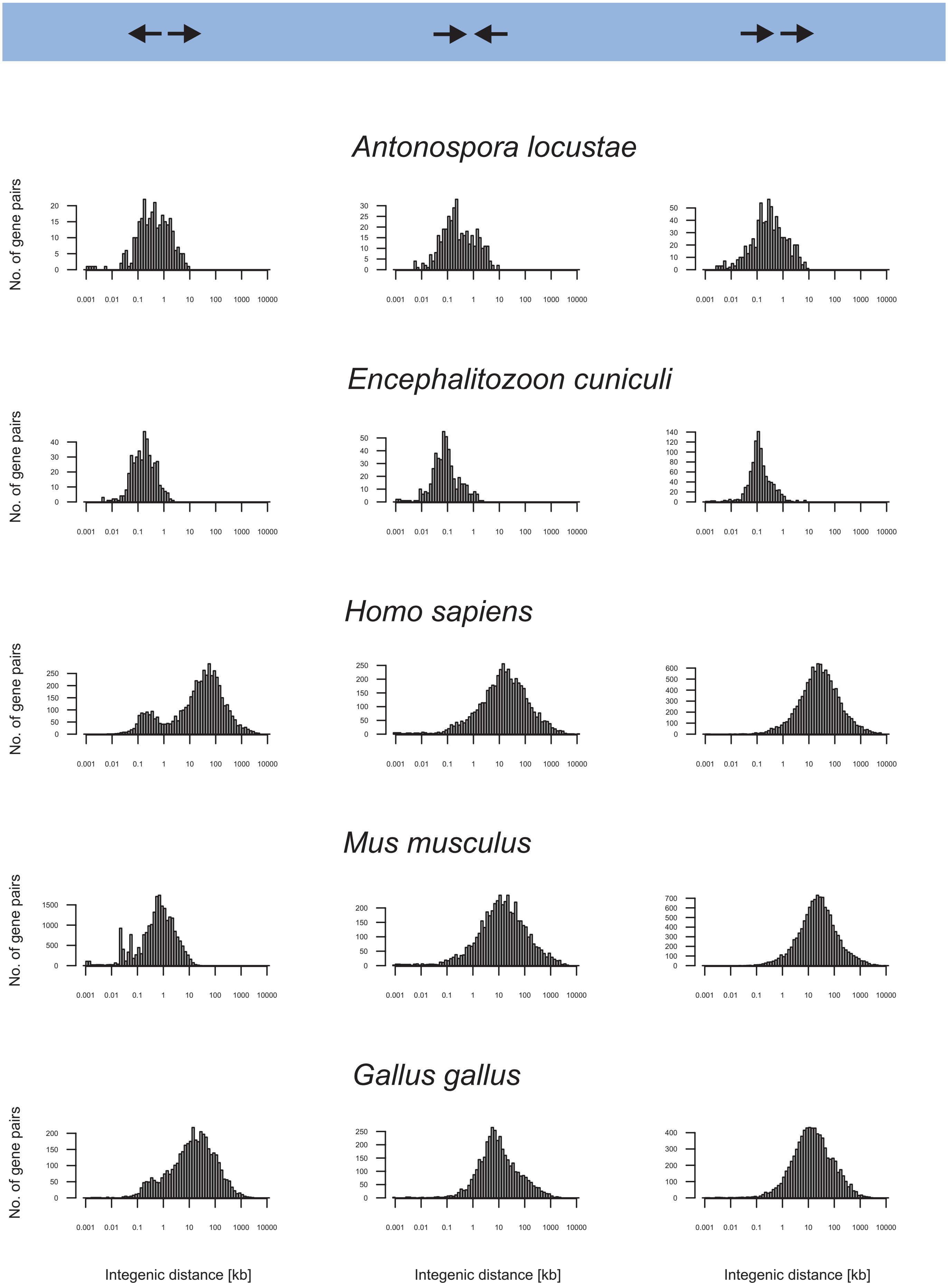

Antonospora locustae
Encephalitozoon cuniculi
Homo sapiens
Mus musculus
Gallus gallus
No. of gene pairs
0.001
0.01
0.1
1
10
100
1000
10000
Integenic distance [kb]
Integenic distance [kb]
Integenic distance [kb]

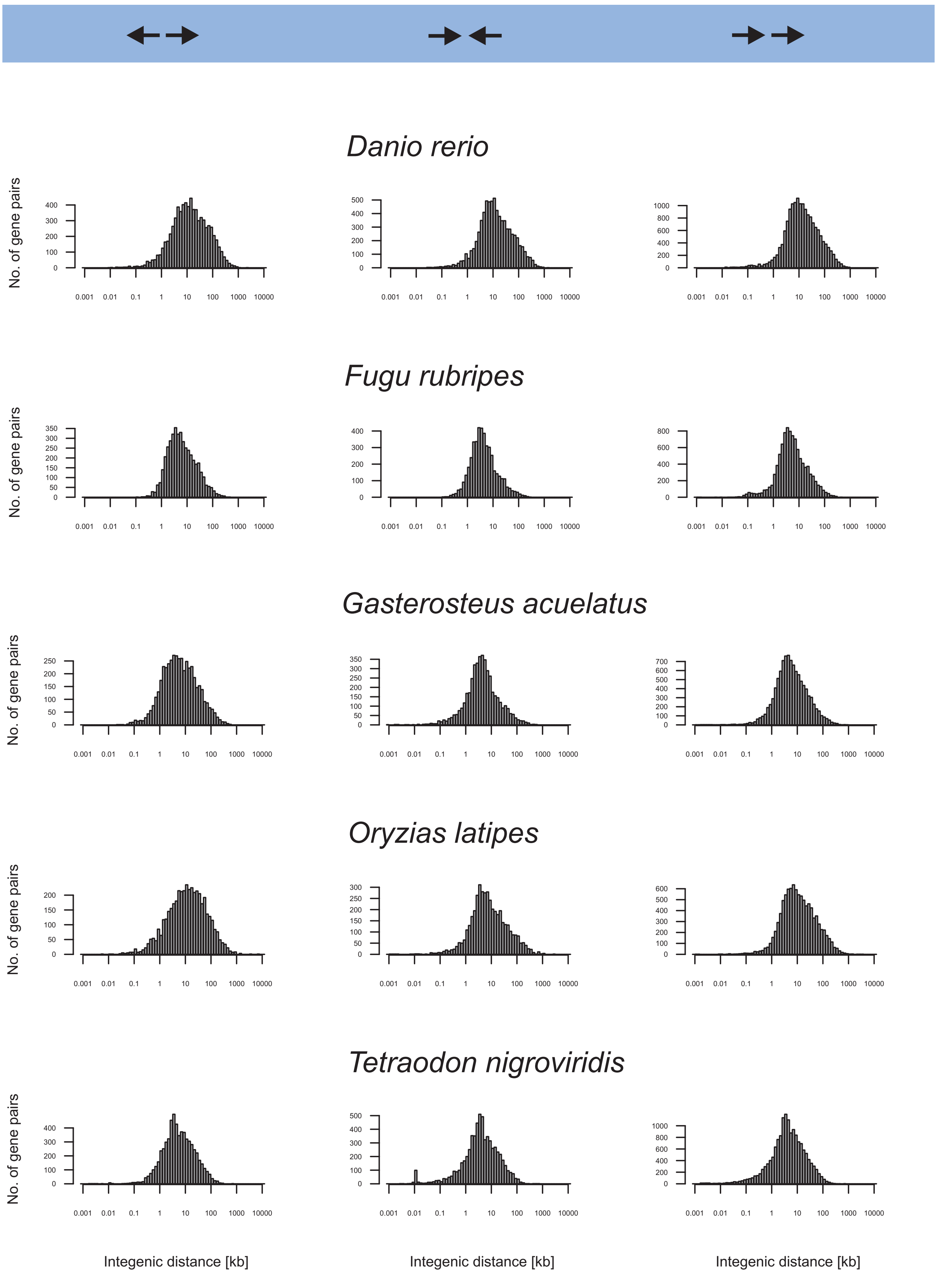
Danio rerio
Fugu rubripes
Gasterosteus acuelatus
Oryzias latipes
Tetraodon nigroviridis
No. of gene pairs
0.001 0.01 0.1 1 10 100 1000 10000
Integenic distance [kb]
Integenic distance [kb]
Integenic distance [kb]

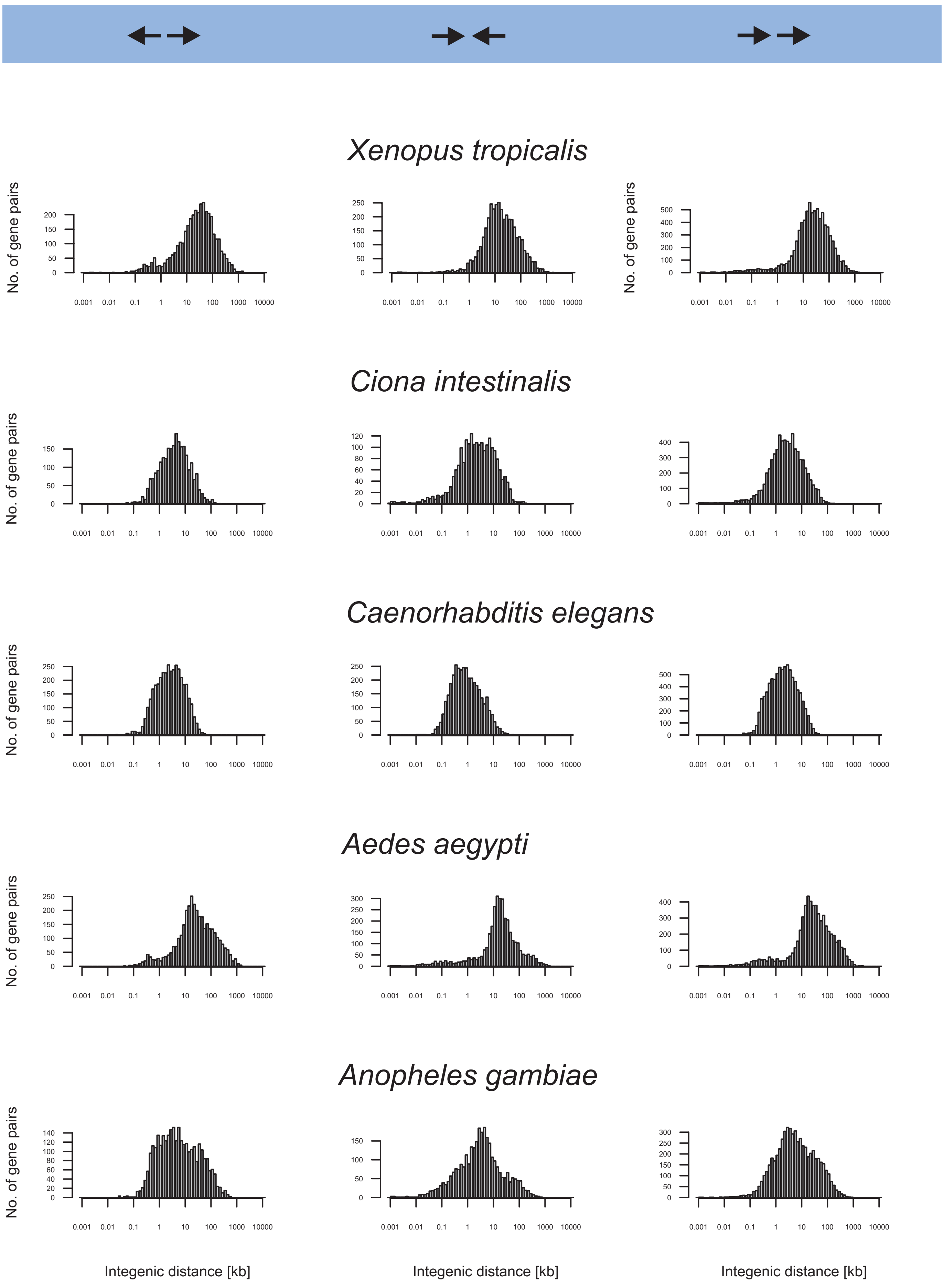
Xenopus tropicalis
Ciona intestinalis
Caenorhabditis elegans
Aedes aegypti
Anopheles gambiae
No. of gene pairs
0.001 0.01 0.1 1 10 100 1000 10000
Integenic distance [kb]
Integenic distance [kb]
Integenic distance [kb]

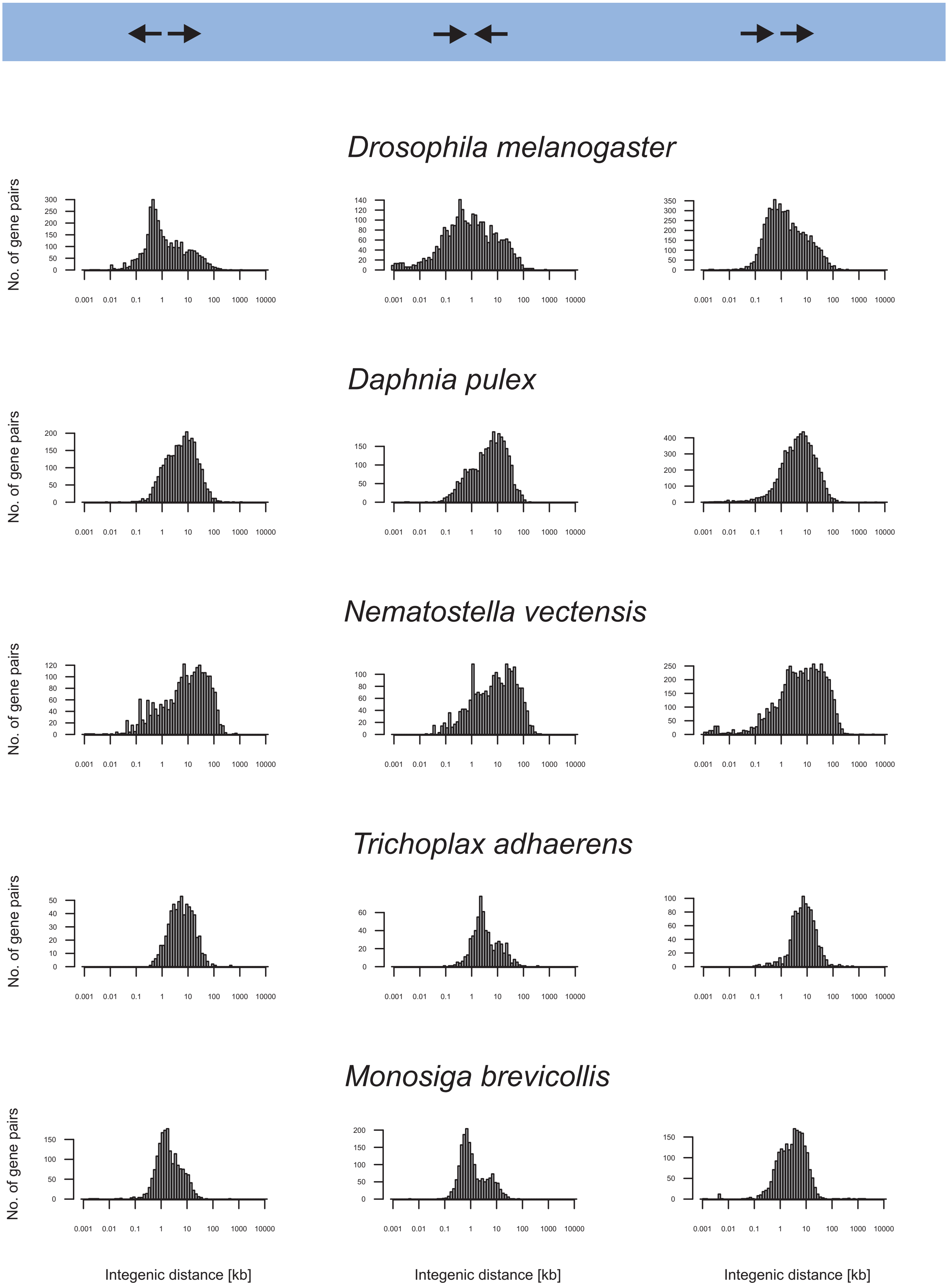
Drosophila melanogaster
Daphnia pulex
Nematostella vectensis
Trichoplax adhaerens
Monosiga brevicollis
No. of gene pairs
0.001 0.01 0.1 1 10 100 1000 10000
Integenic distance [kb]
Integenic distance [kb]
Integenic distance [kb]

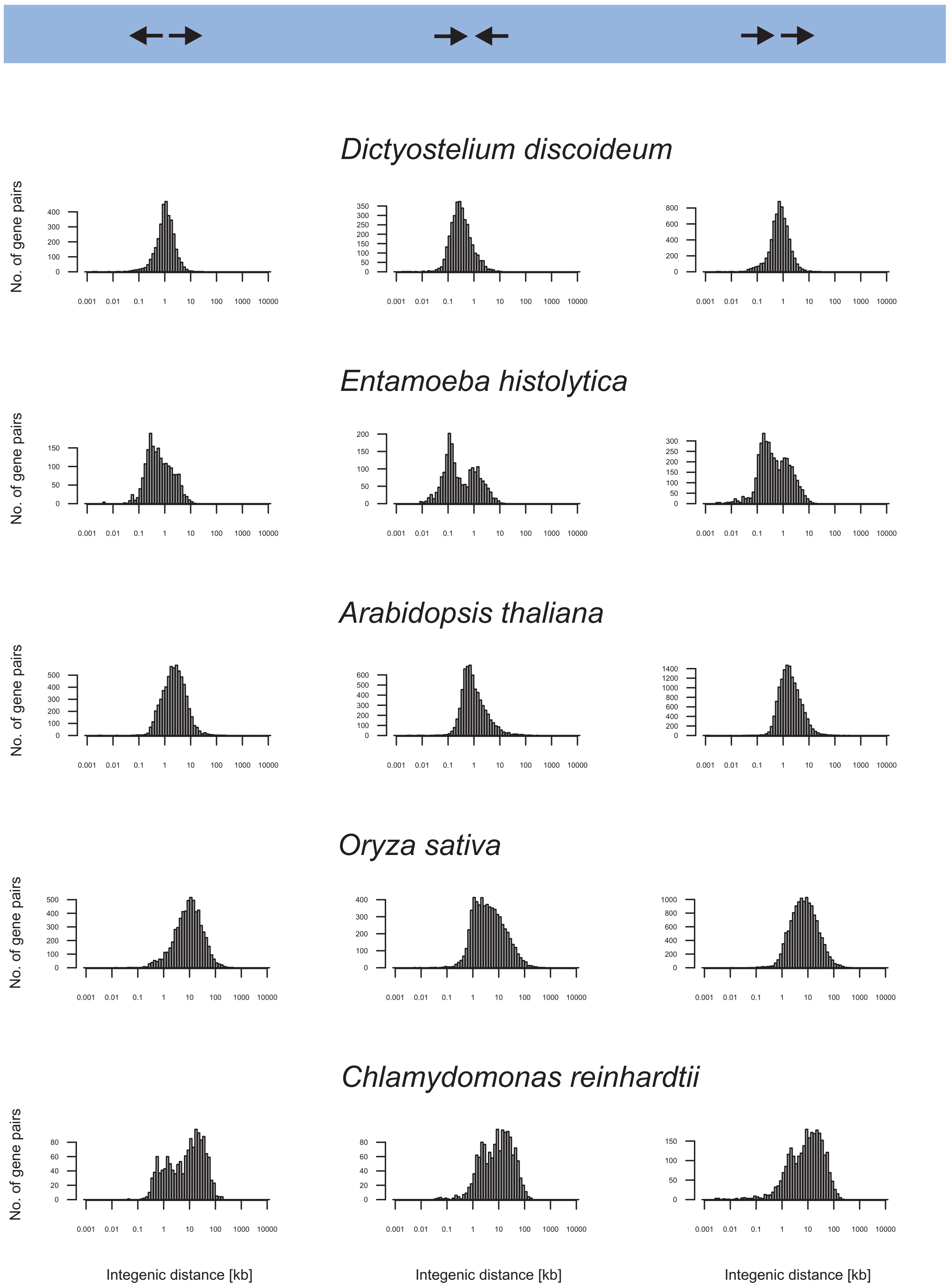
Dictyostelium discoideum
Entamoeba histolytica
Arabidopsis thaliana
Oryza sativa
Chlamydomonas reinhardtii
No. of gene pairs
0.001 0.01 0.1 1 10 100 1000 10000
Integenic distance [kb]
Integenic distance [kb]
Integenic distance [kb]

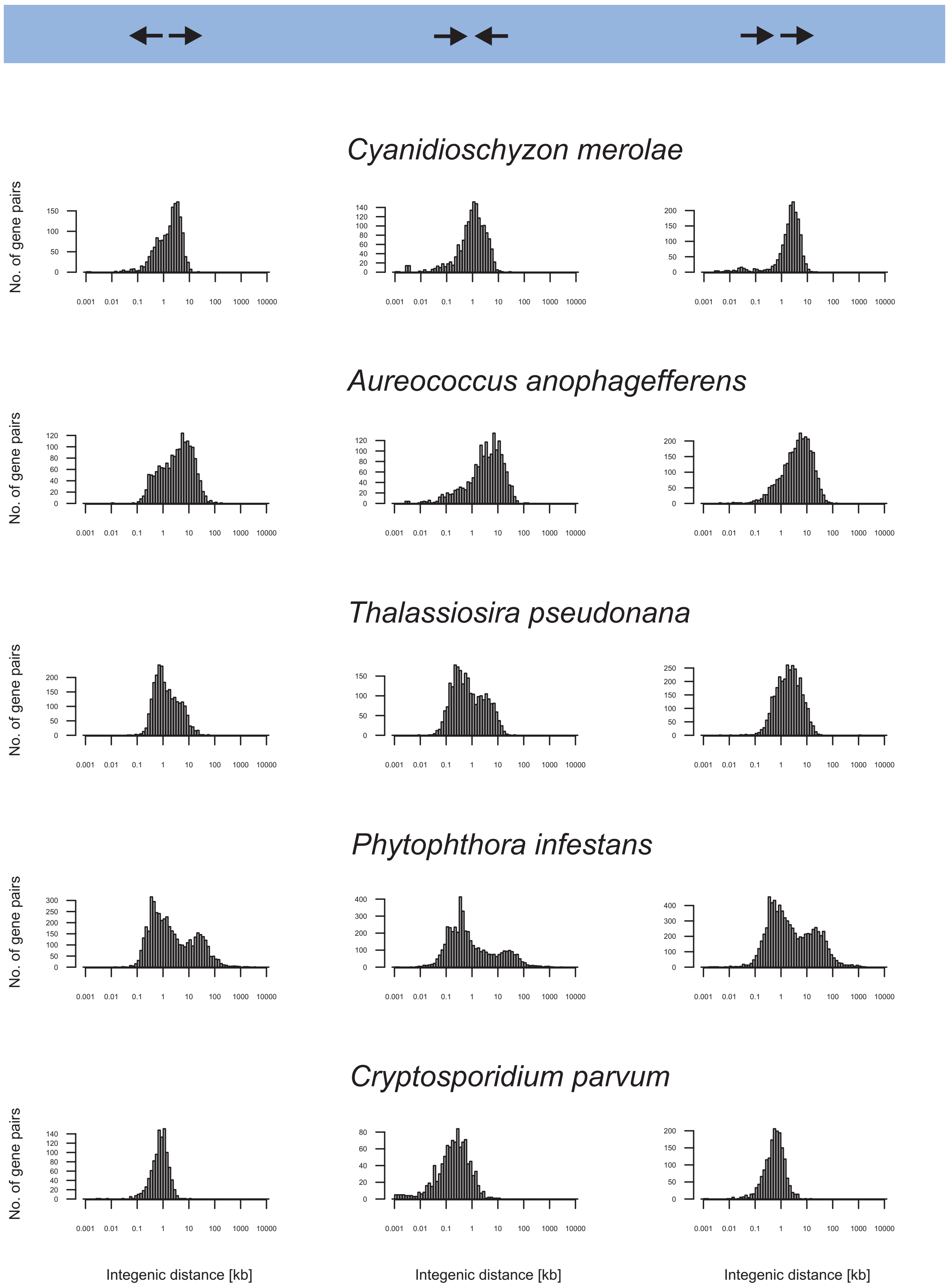
Cyanidioschyzon merolae
Aureococcus anophagefferens
Thalassiosira pseudonana
Phytophthora infestans
Cryptosporidium parvum
No. of gene pairs
0.001 0.01 0.1 1 10 100 1000 10000
Integenic distance [kb]

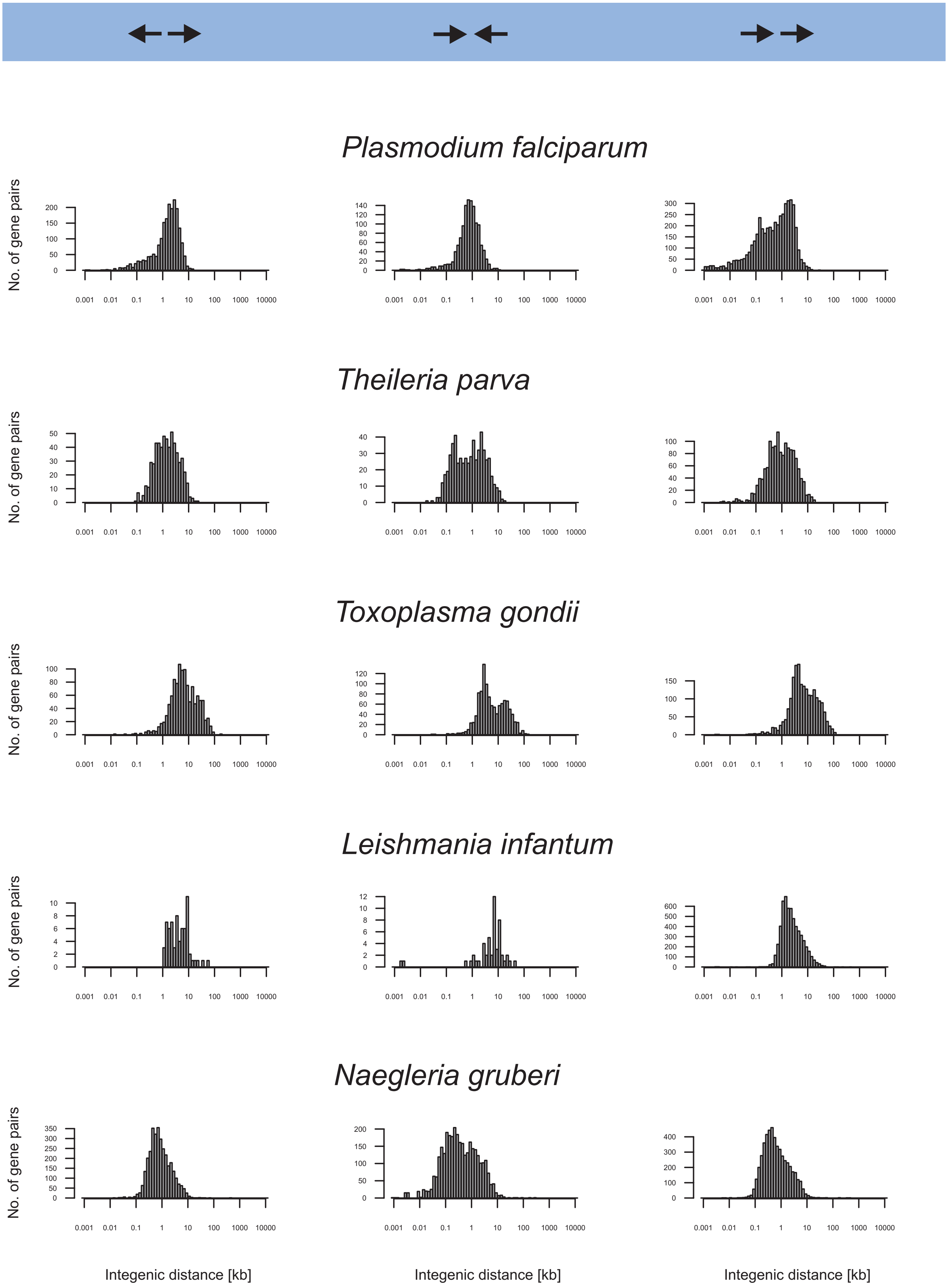
Plasmodium falciparum
Theileria parva
Toxoplasma gondii
Leishmania infantum
Naegleria gruberi
No. of gene pairs
0.001 0.01 0.1 1 10 100 1000 10000
Integenic distance [kb]
Integenic distance [kb]
Integenic distance [kb]

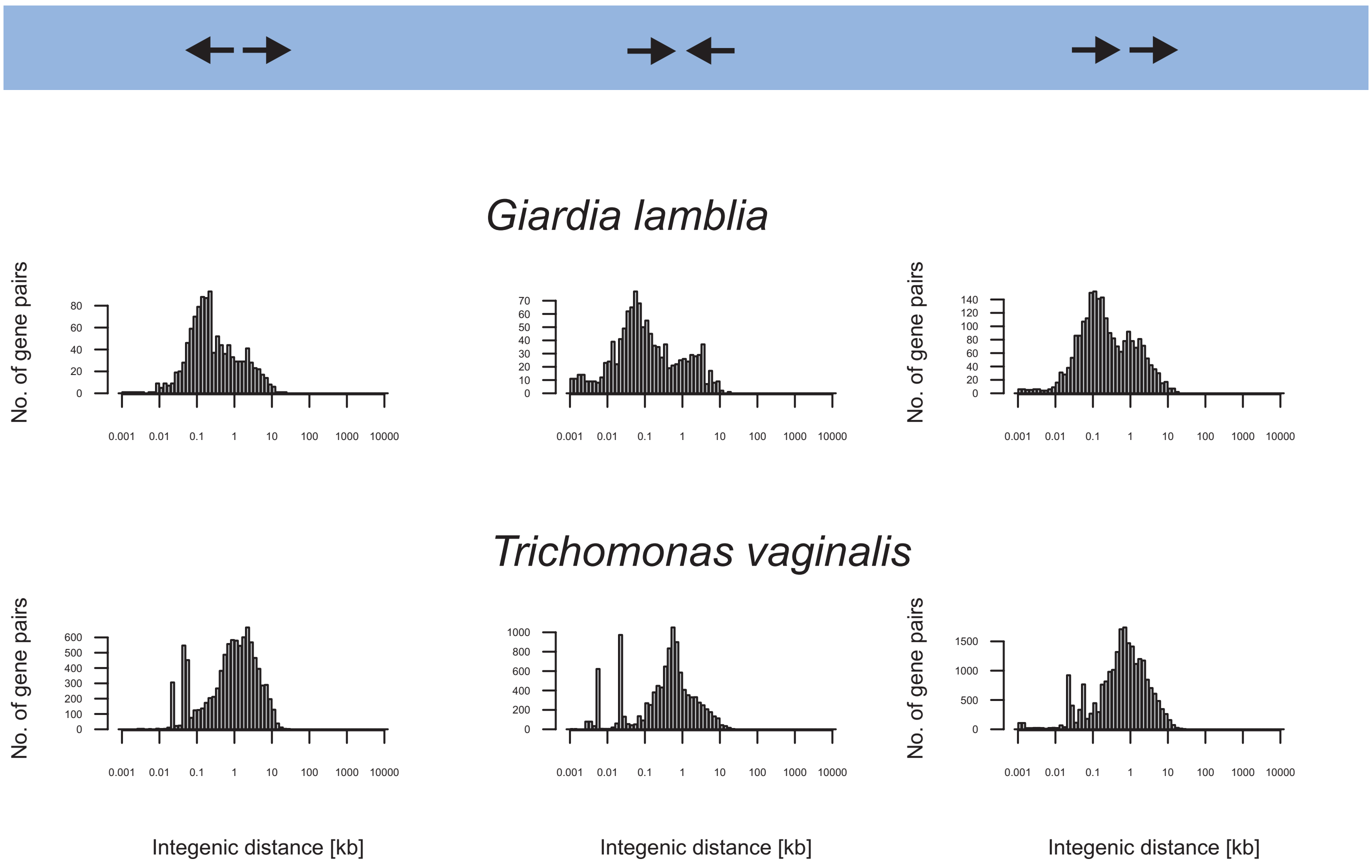
Giardia lamblia
Trichomonas vaginalis
No. of gene pairs
0.001 0.01 0.1 1 10 100 1000 10000
Integenic distance [kb]
