# Supplementary material for: Analysis of Gene Order Conservation in Eukaryotes Identifies Transcriptionally and Functionally Linked Genes
Source: PLoS One. 2010 May 14;5(5):e10654. doi: 10.1371/journal.pone.0010654 (PMC2871058; doi:10.1371/journal.pone.0010654)
Supplement: Table S1 — Sources of genomic sequences, statistics on proteins, relative gene orientation, and clustering using OrthoMCL and Pfam. (0.05 MB PDF) [file pone.0010654.s003.pdf]

Table S1: Sources of genomic sequences, statistics on proteins, relative gene orientation, and clustering using OrthoMCL and Pfam

NOTE: Download date is given where no version could be established. When position information was available from protein sequences or from a correspondent feature table no nucleotide sequences were used to map the proteins. pp stands for protein prediction set release date.

ORGANISM	ABB.		VERSION		Total No. Proteins	Reduced No. Proteins	Relative gene orientation			Clustered by PFAM	Clustered by ORTHOMCL
			P	A			→→	→←	←→		
PEZIZOMYCOTINA											
Aspergillus nidulans	Aspnid	Broad	Release 4		10 701	10 699	4 689	2 899	2 879	6 703	9 019
Aspergillus terreus	Aspter	Broad	pp 06-05-23		10 406	10 406	4 610	2 886	2 891	6 696	9 044
Botrytis cinerea	Botcin	Broad	pp 05-04-10		16 448	16 448	7 375	4 242	4 324	5 874	9 957
Chaetomium globosum	Chaglo	Broad	pp 05-06-27		11 124	11 124	4 760	3 165	3 172	5 739	8 808
Coccidioides immitis	Cocimm	Broad	pp 06-25-01		10 459	10 457	4 765	2 841	2 844	4 895	8 008
Fusarium oxysporum	Fusoxy	Broad	FO2 07-03-27		17 609	17 606	8 176	4 661	4 673	8 906	15 543
Fusarium verticillioides	Fusver	Broad	FV3 06/12		14 241	14 175	6 482	3 827	3 830	7 614	12 402
Gibberella zeae	Gibzea	Broad	FG1 07-03-27		13 321	13 320	5 938	3 681	3 684	7 291	11 155
Histoplasma capsulatum	Hiscap	Broad	pp 06-24-005		9 350	9 349	4 047	2 556	2 557	4 626	7 763
Magnaporthe grisea	Maggri	Broad	v5.0		11 109	11 107	3 865	2 739	2 587	6 012	8 710
Neurospora crassa	Neucra	Broad	v3.0		9 865	9 825	4 287	2 716	2 699	5 306	7 590
Sclerotinia sclerotiorum	Sclscl	Broad	pp 05-10-01		14 522	14 522	6 523	3 980	3 983	5 834	9 732
Stagonospora nodorum	Stanod	Broad	SN15 08-05-15		16 597	16 597	8 228	4 148	4 159	7 096	10 007
Uncinocarpus reesii	Uncree	Broad	pp 06-05-26		7 798	7 798	3 371	2 203	2 201	4 522	6 869
SACCHAROMYCOTINA											
Clavispora lusitaniae	Clalus	Broad	pp 06-01-25		5 941	5 823	2 777	1 518	1 520	3 871	5 035
Candida tropicalis	Cantro	Broad	pp 06-06-12		6 258	6 210	3 048	1 571	1 570	4 222	5 593
Lodderomyces elongisporus	Lodelo	Broad	2007-01-15		5 798	5 743	2 858	1 431	1 428	3 911	5 226
Pichia guilliermondii	Picgui	Broad	pp 06-15-03		5 920	5 832	2 936	1 442	1 445	4 170	5 315
Saccharomyces cerevisiae	Saccer	SGD	2005-02-17		6 652	6 504	2 864	1 814	1 810	4 378	4 853
TAPHRINOMYCOTINA											
Schizosaccharomyces pombe	Schpom	Broad	2008-04-21		4 995	4 987	2 280	1 350	1 351	4 042	4 174
BASIDIOMYCOTA											
Coprinus cinereus	Copcin	Broad	v1.0		13 544	13 544	6 421	3 345	3 351	5 800	9 897
Cryptococcus neoformans	Cryneo	Broad	v3.0		7 299	7 297	2 613	2 232	2 251	4 506	5 642
Puccinia graminis	Pucgra	Broad	pp 07-07-13		20 567	20 566	9 996	5 097	5 107	5 031	15 691

ORGANISM	ABB.		VERSION		Total No. Proteins	Reduced No. Proteins	Relative gene orientation			Clustered by PFAM	Clustered by ORTHOMCL
			P	A			→→	→←	←→		
ZYGOMYCOTA											
Rhizopus oryzae	Rhiory	Broad	RO3 04-09-09		17 413	17 413	8 832	4 254	4 246	8 376	13 837
CHYTRIDIOMYCOTA											
Batrachochytrium dendrobatidis	Batden	Broad	pp 07-01-03		8 863	8 794	4 318	2 198	2 213	4 378	6 618
MICROSPORIDIA											
Antonospora locustae	Antloc	Antonospora locustae DB	2008-04-17	2008-04-17	2 606	2 470	708	430	334	881	1 923
Encephalitozoon cuniculi	Enccun	NCBI	2008-04-18	2008-04-18	1 996	1 945	941	497	496	1 141	1 454
MAMMALS											
Homo sapiens	Homsap	Ensembl	48.36j		46 610	22 481	10 694	5 849	5 856	16 632	19 365
Mus musculus	Musmus	Ensembl	48.37a		39 458	23 193	11 517	5 766	5 774	17 953	20 319
AVES											
Gallus galllus	Galgal	Ensembl	48.2f		22 195	16 597	7 817	4 364	4 364	11 900	13 772
FISHES											
Danio rerio	Danrer	NCBI	2008-02-26		32 191	31 206	16 305	7 440	7 436	22 665	25 944
Fugu rubripes	Fugrub	Ensembl	48.7b		21 974	21 775	9 907	4 560	4 518	16 289	19 985
Gasterosteus aculeatus	Gasacu	Ensembl	46.1d		27 581	20 730	10 356	4 915	4 899	16 456	19 028
Oryzias latipes	Orylat	Ensembl	48.1d		25 107	19 974	9 830	4 617	4 612	15 459	18 313
Tetraodon nigroviridis	Tetnig	Ensembl	48.1j		28 002	27 932	15 426	6 239	6 240	16 453	19 426
AMPHIBIA											
Xenopus tropicalis	Xentro	JGI	v4.1		29 843	18 811	7 831	4 124	4 055	14 674	16 403
UROCHORDATA											
Ciona intestinalis	Cioint	Ensembl	46.2g		19 858	13 896	6 819	2 720	2 729	7 797	9 890
NEMATODA											
Brugia malayi	Brumal	NCBI	2007-08-31	2007-08-31	379	278	24	5	3	196	249
Caenorhabditis elegans	Caeele	NCBI	2008-02-25		16 429	16 303	8 474	3 912	3 911	9 538	11 768

ORGANISM	ABB.		VERSION		Total No. Proteins	Reduced No. Proteins	Relative gene orientation			Clustered by PFAM	Clustered by ORTHOMCL
			P	A			→→	→←	←→		
INSECTS											
Aedes aegypti	`Aedaeg`	Ensembl	44.1a		16 789	15 324	6 091	3 902	3 719	10 573	12 981
Anopheles gambiae	`Anogam`	Ensembl	46.3i		13 133	12 359	5 936	3 208	3 209	8 658	10 514
Drosophila melanogaster	`Dromel`	Ensembl	46.43b		19 837	13 622	5 888	3 860	3 862	9 370	10 423
CRUSTACEA											
Daphnia pulex	`Dappul`	JGI	v1.0	v1.0	22 636	14 776	6 521	3 201	3 204	6 668	12 174
CNIDARIA											
Nematostella vectensis	`Nemvec`	JGI	v1.0	v1.0	115 414	17 131	5 690	4 040	3 937	1 903	14 514
PLACOZOA											
Trichoplax adhaerens	`Triadh`	JGI	v1.0	v1.0	2 360	2 283	1 023	626	628	1 645	1 945
CHOANOFLAGELLATA											
Monosiga brevicollis	`Monbre`	JGI	v1.0	v1.0	24 784	6 191	2 124	1 936	1 961	3 940	4 584
MYCETOZOA											
Dictyostelium discoideum	`Dicdis`	DictyBase/NCBI	2007-01-23	2008-02-20	13 345	13 343	6 655	3 342	3 340	6 868	9 107
ENTAMOEBIDAE											
Entamoeba histolytica	`Enthis`	Sanger	2007-08-31		17 203	9 641	4 100	1 977	1 952	4 191	8 013
STREPTHOPHYTA											
Arabidopsis thaliana	`Aratha`	NCBI	2005-11-16		31 711	26 812	14 060	6 373	6 374	20 040	22 596
Oryza sativa	`Orysat`	NCBI	2008-03-04		26 777	26 674	13 559	6 552	6 551	17 646	19 028
GREEN ALGAE											
Chlamydomonas reinhardtii	`Chlrei`	JGI	v3.0	v3.0	9 411	6 419	2 772	1 503	1 491	3 280	4 432
Volvox carteri	`Volcar`	JGI	v1.0	v1.0	5 605	4 304	1 787	1 113	1 113	1 828	3 594
RED ALGAE											
Cyanidioschyzon merolae	`Cyamer`	Cyanidioschyzon merolae DB	2008-02-26		5 014	4 953	1 670	1 606	1 601	3 363	3 497

ORGANISM	ABB.		VERSION		Total No. Proteins	Reduced No. Proteins	Relative gene orientation			Clustered by PFAM	Clustered by ORTHOMCL
			P	A			→→	→←	←→		
PELAGOPHYTES											
Aureococcus anophagefferens	Aurano	JGI	v1.0	v1.0	8 292	7 215	2 941	1 793	1 800	4 702	5 635
DIATOMEA											
Thalassiosira pseudonana	Thapse	JGI	v3.0	v3.0	8 542	8 227	3 187	2 505	2 508	4 412	5 782
OOMYCETES											
Phytophthora infestans	Phyinf	Broad	pp 09-09-04		17 898	17 892	7 833	4 682	4 646	7 974	13 563
APICOMPLEXA											
Cryptosporidium parvum	Crypar	NCBI	2008-02-26		3 805	3 798	1 715	1 036	1 039	1 905	2 202
Plasmodium falciparum	Plafal	Plasmodb	Release 5.3		27 917	9 173	4 816	2 170	2 169	3 519	6 239
Theileria parva	Thepar	TIGR	2007-08-30	2007-08-30	3 271	2 608	1 422	587	590	1 286	1 702
Toxoplasma gondii	Toxgon	Toxodb	v4.2	v4.2	4 579	4 514	2 091	1 167	1 168	1 943	2 478
EUGLENOZOA											
Leishmania infantum	Leiinf	Sanger	V5.2	V5.2	6 802	5 906	5 747	53	70	2 993	3 044
HETEROLOBOSEA											
Naegleria gruberi	Naegru	JGI	v1.0	v1.0	52 330	12 511	5 078	3 446	3 447	6 598	9 304
DIPLOMANDIDA											
Giardia lamblia	Gialam	GiardiaDB	Release 1.1		4 889	4 790	2 275	1 240	1 237	2 188	2 699
PARABASALIDEA											
Trichomonas vaginalis	Trivag	TrichDB	Release 1		59 672	59 637	22 295	10 860	9 475	13 271	48 480
