# Supplementary material for: Analysis of Gene Order Conservation in Eukaryotes Identifies Transcriptionally and Functionally Linked Genes
Source: PLoS One. 2010 May 14;5(5):e10654. doi: 10.1371/journal.pone.0010654 (PMC2871058; doi:10.1371/journal.pone.0010654)
Supplement: Table S3 — Intergenic distances characteristic of different species. (0.01 MB PDF) [file pone.0010654.s005.pdf]

Table S3: Intergenic distances

ORGANISM	MEAN	MEDIAN	SD
Aspergillus nidulans	1 121	752	1 116
Aspergillus terreus	1 110	771	1 128
Botrytis cinerea	1 322	959	1 220
Chaetomium globosum	1 338	984	1 289
Coccidioides immitis	1 177	719	1 827
Fusarium oxysporum	2 039	1 157	2 744
Fusarium verticillioides	1 507	1 017	1 548
Gibberella zeae	1 229	832	1 263
Histoplasma capsulatum	1 833	1 085	2 233
Magnaporthe grisea	1 352	978	1 231
Neurospora crassa	2 006	1 349	2 814
Sclerotinia sclerotiorum	1 295	974	1 082
Stagonospora nodorum	829	553	1 572
Uncinocarpus reesii	1 220	841	2 163
Clavispora lusitaniae	780	575	776
Candida tropicalis	887	436	1 277
Lodderomyces elongisporus	1 174	683	1 459
Pichia guilliermondii	427	236	586
Saccharomyces cerevisiae	540	353	655
Schizosaccharomyces pombe	3 459	722	45 495
Coprinus cinereus	976	672	1 038
Cryptococcus neoformans	852	529	2 781
Puccinia graminis	2 680	1 479	3 556
Rhizopus oryzae	1 425	957	1 629
Batrachochytrium dendrobatidis	886	565	1 131
Antonospora locustae	610	238	945
Encephalitozoon cuniculi	180	91	330
Homo sapiens	87 820	18 115	332 093
Mus musculus	75 692	16 201	235 307
Gallus gallus	43 790	9 728	114 376
Danio rerio	30 646	9 805	59 969
Fugu rubripes	9 798	3 735	18 826
Gasterosteus aculeatus	12 752	3 976	28 709
Oryzias latipes	28 293	7 590	82 460
Tetraodon nigroviridis	9 303	3 426	17 904
Xenopus tropicalis	45 028	17 957	80 920
Ciona intestinalis	5 592	2 233	9 898
Brugia malayi	2 664	1 509	3 128
Caenorhabditis elegans	3 075	1 421	4 542

ORGANISM	MEAN	MEDIAN	SD
Aedes aegypti	57 124	17 869	111 932
Anopheles gambiae	17 397	3 630	39 534
Drosophila melanogaster	5 565	797	16 480
Daphnia pulex	20 627	4 628	111 722
Nematostella vectensis	36 219	6 409	124 125
Trichoplax adhaerens	11 163	4 503	119 671
Monosiga brevicollis	14 176	1 476	148 448
Dictyostelium discoideum	792	555	940
Entamoeba histolytica	907	321	2 548
Arabidopsis thaliana	2 533	1 266	5 801
Oryza sativa	11 173	5 191	19 068
Chlamydomonas reinhardtii	25 460	8 075	145 266
Volvox carteri	51 297	9 829	206 498
Cyanidioschyzon merolae	2 011	1 433	11 521
Aureococus anophagefferens	16 471	3 684	114 663
Thalassiosira pseudonana	2 783	1 047	18 830
Phytophthora infestans	21 468	1 012	156 548
Cryptosporidium parvum	585	438	631
Plasmodium falciparum	1 153	705	1 419
Theileria parva	1 514	811	1 915
Toxoplasma gondii	10 472	4 581	28 930
Leishmania infantum	3 211	1 684	7 015
Naegleria gruberi	2 332	471	26 625
Giardia lamblia	646	127	1 352
Trichomonas vaginalis	1 154	514	1 737
